# Opposing effects of apoE2 and apoE4 on microglial activation and lipid metabolism in response to demyelination

**DOI:** 10.1186/s13024-022-00577-1

**Published:** 2022-11-23

**Authors:** Na Wang, Minghui Wang, Suren Jeevaratnam, Cassandra Rosenberg, Tadafumi C. Ikezu, Francis Shue, Sydney V. Doss, Alla Alnobani, Yuka A. Martens, Melissa Wren, Yan W. Asmann, Bin Zhang, Guojun Bu, Chia-Chen Liu

**Affiliations:** 1 Department of Neuroscience, Mayo Clinic, Jacksonville, FL 32224 USA; 2 Department of Genetics and Genomic Sciences, Icahn School of Medicine at Mount Sinai, New York, NY 10029 USA; 3 Division of Biomedical Statistics and Informatics, Department of Health Sciences Research, Mayo Clinic, Jacksonville, FL 32224 USA

**Keywords:** Microglia, apoE isoforms, Demyelination, Lipid droplets, Remyelination

## Abstract

**Background:**

Abnormal lipid accumulation has been recognized as a key element of immune dysregulation in microglia whose dysfunction contributes to neurodegenerative diseases. Microglia play essential roles in the clearance of lipid-rich cellular debris upon myelin damage or demyelination, a common pathogenic event in neuronal disorders. Apolipoprotein E (apoE) plays a pivotal role in brain lipid homeostasis; however, the apoE isoform-dependent mechanisms regulating microglial response upon demyelination remain unclear.

**Methods:**

To determine how apoE isoforms impact microglial response to myelin damage, 2-month-old apoE2-, apoE3-, and apoE4-targeted replacement (TR) mice were fed with normal diet (CTL) or 0.2% cuprizone (CPZ) diet for four weeks to induce demyelination in the brain. To examine the effects on subsequent remyelination, the cuprizone diet was switched back to regular chow for an additional two weeks. After treatment, brains were collected and subjected to immunohistochemical and biochemical analyses to assess the myelination status, microglial responses, and their capacity for myelin debris clearance. Bulk RNA sequencing was performed on the corpus callosum (CC) to address the molecular mechanisms underpinning apoE-mediated microglial activation upon demyelination.

**Results:**

We demonstrate dramatic isoform-dependent differences in the activation and function of microglia upon cuprizone-induced demyelination. ApoE2 microglia were hyperactive and more efficient in clearing lipid-rich myelin debris, whereas apoE4 microglia displayed a less activated phenotype with reduced clearance efficiency, compared with apoE3 microglia. Transcriptomic profiling revealed that key molecules known to modulate microglial functions had differential expression patterns in an apoE isoform-dependent manner. Importantly, apoE4 microglia had excessive buildup of lipid droplets, consistent with an impairment in lipid metabolism, whereas apoE2 microglia displayed a superior ability to metabolize myelin enriched lipids. Further, apoE2-TR mice had a greater extent of remyelination; whereas remyelination was compromised in apoE4-TR mice.

**Conclusions:**

Our findings provide critical mechanistic insights into how apoE isoforms differentially regulate microglial function and the maintenance of myelin dynamics, which may inform novel therapeutic avenues for targeting microglial dysfunctions in neurodegenerative diseases.

**Supplementary Information:**

The online version contains supplementary material available at 10.1186/s13024-022-00577-1.

## Background

As the resident immune cells in the central nervous system (CNS), microglia maintain brain homeostasis and orchestrate inflammatory responses to injury [1–5]. Microglia are highly motile cells that constantly survey the brain parenchyma to eliminate cellular debris and dysfunctional synapses [6, 7]. During aging and pathological challenges, microglia transition to reactive states leading to morphological changes with enhanced phagocytic activity and cytokine production [8, 9]. Microglia are the first responders for clearing cellular debris generated from damaged myelin sheaths, a lipid-rich substance essential for efficient electrical communication between neurons [8, 10, 11]. Cumulative evidence supports that the lipid metabolism and immune response of microglia are critical in the debris clearance of myelin damage, a common feature of neuronal disorders [12–22]. However, extensive myelin degradation may overwhelm the efflux capacity of microglia, leading to the accumulation of fatty acids and lipid-rich debris in lipid droplets upon demyelination [17, 23]. Defective cholesterol transport or efflux by apolipoproteins may exacerbate pathogenic lipid accumulation in microglia, resulting in microglial dysfunction during brain aging and disease progression [15, 24]. In addition, the reduced clearance of damaged myelin by microglia is suggested to account for the excessive accumulation of myelin debris, which impairs the rate of myelin regeneration and the quality of tissue repair [13, 25, 26]. Microglial dysfunction and myelin abnormalities play important pathogenic roles in neurodegenerative disease such as Alzheimer’s disease (AD) [9, 27–30]. Thus, defining the molecular mechanisms by which microglia mediate lipid metabolism as it relates to myelin debris clearance and remyelination may guide therapeutic strategy for neurological disorders.

ApoE mediates lipid transport between glia and neurons, as well as other CNS cells, to aid their normal function and support tissue repair [31, 32]. Human *APOE* possesses three major allelic variants which result in isoforms that differ at two amino acid residues, 112 and 158 (ApoE2: Cys112, Cys158; ApoE3: Cys112, Arg158; ApoE4: Arg112, Arg158) [31, 33]. The structural and functional differences between apoE isoforms have been shown to confer differential susceptibility to neurodegenerative diseases such as AD and LBD, with *APOE4* increasing the risk while *APOE2* is protective compared with the more common *APOE3* allele [32–35]. ApoE is known to be primarily secreted by astrocytes; however, studies suggest that elevated microglial apoE plays a critical role in regulating the innate immune response during aging and neurodegeneration [36–40]. Importantly, apoE was identified as one of the top up-regulated markers in subpopulations of microglia, including disease associated microglia (DAM) and activated response microglia (ARM) [39, 41–44]. Moreover, several studies demonstrated that apoE, along with its associated phospholipids, is a ligand for the Triggering Receptor Expressed on Myeloid cells-2 (TREM2), a key gene expressed by microglia [45–47]. Thus, understanding the impact of apoE isoforms on microglial response and functions, in the context of neuronal injury such as demyelination, may facilitate the development of therapeutic strategies for the treatment of neurodegenerative diseases.

To investigate apoE-mediated microglial responses after tissue injury, we treated apoE-TR mice with cuprizone (CPZ), a copper-chelator that induces CNS demyelination. Using this model, we demonstrate that apoE isoforms differentially influenced microglial activation, proliferation, morphological changes, as well as transcriptomic signatures in response to myelin damage. Specifically, apoE2 microglia were hyperactive and had superior functions in lipid metabolism and phagocytic ability, while apoE4 microglia appear to be less activated and exhibited functional deficits. Such apoE isoform-dependent microglial responses may play a critical role in regulating the clearance of myelin debris and associated lipid metabolism which influences tissue repair. In sum, our study uncovers a mechanistic link between apoE isoforms and dysregulation of microglial function in vivo, implying that apoE-mediated microglial dysfunction may contribute to the development of neuronal disorders.

## Materials and methods

### Animals

ApoE2-, apoE3- and apoE4-TR mice, expressing human *APOE* gene alleles driven by the endogenous murine *Apoe* promoter, were obtained from Taconic. Animals were housed under controlled conditions of temperature and lighting and given free access to food and water. Both male and female mice were used in the study. The animal number and information used in each experiment are described in detail within the figure legends. In the demyelination study, 2- month-old mice in control and CPZ-treated groups were fed with regular rodent chow or chow supplemented with 0.2% cuprizone (Envigo, TD.140801) for four weeks. To assess remyelination, the cuprizone diet was replaced after four weeks with regular rodent chow for an additional two weeks.

### Immunohistochemistry

After perfusion with phosphate buffered solution (PBS), mouse brains were harvested and fixed in 10% neutralized formalin for histological analysis. Immunohistochemistry was performed as previously described [48–50]. Briefly, paraffin-embedded Sects. (5 μm thick) were dewaxed, rehydrated, and subjected to heat induced antigen retrieval by steaming in 10 mM sodium citrate (pH 6.0) for 30 min. This was followed by immersing tissue sections in 0.3% H_2_O_2_ for 10 min to block endogenous peroxidases. Tissue sections were incubated with primary antibody at 4 °C overnight followed by a corresponding secondary antibody for 1 h at room temperature (RT). The following primary antibodies were used in the study: anti-GFAP antibody (1:1000, Millipore, MAB360); anti-myelin basic protein (MBP) antibody (1:200, Millipore, MAB382); anti-degraded myelin basic protein (dMBP) antibody (1:100, Millipore, AB5864); anti-Perilipin-2 (Plin2) antibody (1:200, Novus, NB110-40877); anti-ionized calcium-binding adaptor molecule 1 (Iba-1) antibody (1:1000, Wako, 19–19741); anti-Iba-1 antibody (1:200, Wako, 011–27991). For some experiments, frozen Sects. (40 μm thick) were subjected to immunofluorescence staining. The following primary antibodies were used for the staining of frozen sections: anti-Ki67 antibody (1:200, Abcam, Ab16667); anti-Iba-1 antibody (1:300, Wako, 011–27991); anti-CD68 antibody (1:100, Bio-Rad, MCA1957); anti-Iba-1 antibody (1:300, CST, 011–27991); anti-CLDN5 antibody (1:200, Invitrogen, #35–2500); anti-Glut1 antibody (1:200, Abcam, Ab15309).

### RNA isolation and quantitative real-time PCR

Corpus callosum (CC) was dissected from whole brains under a light microscope. Trizol (Invitrogen) and Direct-zol RNA MiniPrep kit (Zymo Research) were used for total RNA extraction according to the manufacturers’ instructions. RNA concentration was measured by a NanoDrop 1000 device (Thermo Fisher Scientific). Complementary DNA was synthesized using the ReverTra Ace qPCR RT Kit (TOYOBO, FSQ-101). Quantitative real-time PCR (q-PCR) analysis was performed using the LightCycler 480 SYBR Green/Master system. Gene expression in the CC brain region of apoE-TR mice fed with either CPZ-containing or normal chow was analyzed. GAPDH was used as the q-PCR internal control. The ΔCt method was used to determine differences in the gene expression levels between CPZ-fed mice and age-matched apoE-TR mice fed with normal chow, for each isoform. Primers used for amplifying target genes are listed in Table 1:

**Table 1:** Primer pairs used for quantitative real-time PCR

Gene	Forward primer	Reverse primer
*Gapdh*	5’- GGTGAAGGTCGGTGTGAACG-3′	5’- CTCGCTCCTGGAAGATGGTG-3′
*Csf1*	5’- AGTATTGCCAAGGAGGTGTCAG-3′,	5’- ATCTGGCATGAAGTCTCCATTT-3′
*Csf1r*	5’- GCAGTACCACCATCCACTTGTA-3′	5’- GTGAGACACTGTCCTTCAGTGC-3’
*Itgax*	5’-ATGGAGCCTCAAGACAGGAC-3’	5’-GGATCTGGGATGCTGAAATC-3’
*Mmp2*	5’-GCTGTATTCCCGACCGTTGA-3’	5’-TGGTCCGCGTAAAGTATGGG-3’
*Mmp12*	5’-CTGCTCCCATGAATGACAGTG-3’	5’-AGTTGCTTCTAGCCCAAAGAAC-3’
*Tnf-α*	5’-ATGAGCACAGAAAGCATGATCCGCG-3’	5’-CCCTTCACAGAGCAATGACTCCAAA -3’
*Il-1β*	5’- ATGGCAACTGTTCCTGAACTCAACT-3’	5’- AGGACAGGTATAGATTCTTTCCTTT-3’
*CD68*	5’- TGTCTGATCTTGCTAGGACCG-3’	5’- GAGAGTAACGGCCTTTTTGTGA-3’
*Axl*	5’- GGAGGAGCCTGAGGACAAAGC-3’	5’-GACAGCATCTTGAAGCCAGAGTAGG-3’
*Cr3*	5’- CGACACCATCGCATCTAA-3’	5’- TCCCTGAACATCACCACC-3’
*Lpl*	5’- TTCCAGCCAGGATGCAACA-3’	5’- GGTCCACGTCTCCGAGTCC-3’
*Apoc1*	5’-TTCAGTTCGTGTGTGGACCGA-3’	5’- ATCCACAATGCCTGTCTGAGG-3’
*Trem2*	5’- TCATAGGGGCAAGACACCT-3’	5’- GCTGCTCATCTTACTCTTTGTC-3’
*Tyrobp*	5’- GAGTGACACTTTCCCAAGATGC-3’	5’- CCTTGACCTCGGGAGACCA-3’
Aim2	5’- GTCACCAGTTCCTCAGTTGTG-3’	5’- CACCTCCATTGTCCCTGTTTTAT-3’
*C3*	5’- CCAGCTCCCCATTAGCTCTG-3’	5’- GCACTTGCCTCTTTAGGAAGTC-3’
*Clec7a*	5’- GACTTCAGCACTCAAGACATCC-3’	5’- TTGTGTCGCCAAAATGCTAGG-3’
*Serpina3n*	5’- ATTTGTCCCAATGTCTGCGAA-3’	5’- TGGCTATCTTGGCTATAAAGGGG-3’
*Btk*	5’- AAGAAGCGCCTGTTTCTCTTG-3’	5’- GGTACGGGAACCTTTCAATGAT-3’
*Cst7*	5’- GGAGCTGTACTTGCCGAGC-3’	5’- CATGGGTGTCAGAAGTTAGGC-3’
*Ccl2*	5’- ATTCTGTGACCATCCCCTCAT-3’	5’- TGTATGTGCCTCTGAACCCAC-3’
*Gpnmb*	5’- TGCCAAGCGATTTCGTGATGT-3’	5’- GCCACGTAATTGGTTGTGCTC-3’
B2m	5’- TTCTGGTGCTTGTCTCACTGA-3’	5’- CAGTATGTTCGGCTTCCCATTC-3’
*Ctsd*	5’- GCTTCCGGTCTTTGACAACCT-3’	5’- CACCAAGCATTAGTTCTCCTCC-3’

### Western blotting

Following dissection, the corpus callosum was lysed with RIPA buffer (Thermo Fisher, 89900), and total protein content was quantified by BCA assay (Pierce BCA Protein Assay Kit, 23225). Western blotting was performed as previously described [51]. Briefly, protein (40 µg) was loaded onto 10% SDS-PAGE gel and transferred onto PVDF membranes (Millipore). The membrane was incubated with primary antibody overnight at 4 °C, followed by corresponding HRP-conjugated secondary antibody. The protein bands were visualized using ECL detection system (Pierce) and exposed to film. Anti-tubulin (Millipore, MABT205) and anti-Iba1 (Wako, 016–20001) antibodies were used in this study. Immuno-reactive bands were quantified using Image J.

### RNA sequencing and analysis

RNA isolation was performed as described above. Paired-end RNA-seq data was generated with the Illumina HiSeq 4000 platform. Raw sequencing reads were aligned to mouse mm10 genome using star aligner [52] (version 2.4.0g1). Following read alignment, “featureCounts” [53] (v1.6.3) was used to quantify the gene expression at the gene level based on the GENCODE gene model. Genes identified with 1 count per million (CPM) reads in at least 1 sample were considered expressed and hence retained for further analysis. The read count data were normalized using the trimmed mean of M-values normalization (TMM) method [54] in order to adjust for sequencing library size difference. Differential expression analysis between CPZ-treated mice and control mice within each apoE isoform was performed using linear model analysis implemented in R package “limma”. Meanwhile, differential response to CPZ treatment among apoE isoforms were also computed using “limma”. RNA integrity number (RIN) was included as a covariate in the differential expression analysis. Genes with Benjamini-Hochberg’s false discovery rate (FDR) [55] ≤ 0.05, and fold change (FC) ≥ 1.2 were considered significant.

To model the gene–gene co-expression relationship, we applied multiscale embedded gene co-expression network analysis (MEGENA) [56], a multiscale approach that allows for identification of overlapping gene modules, as well as individual gene–gene interactions. Briefly, Pearson correlation coefficients were first computed for all gene pairs in either CPZ-treated mice or control mice. The gene pairs with significant correlation (FDR adjusted P value less than 0.05) were ranked and iteratively tested for planarity to grow a Planar Filtered Network (PFN). Then multiscale clustering analysis was conducted to identify co-expression modules under the default parameter setting of the MEGENA package (version 1.3.4–1).

### Statistical analysis

A one-way ANOVA with Tukey’s multiple comparisons test was used to assess the differences between three groups. A two-way ANOVA with Tukey’s multiple comparisons test was used to assess the differences between three groups, in which both genotype and treatment group were taken into consideration. A *P* value of < 0.05 was considered significant. Data in figures are presented as mean ± standard error of the mean (SEM). All statistical tests were performed using GraphPad Prism version 6.0 (SCR_002798).

## Results

### ApoE isoforms differentially regulate the clearance of myelin debris in apoE-TR mice upon cuprizone-induced demyelination

**Fig. 1:**
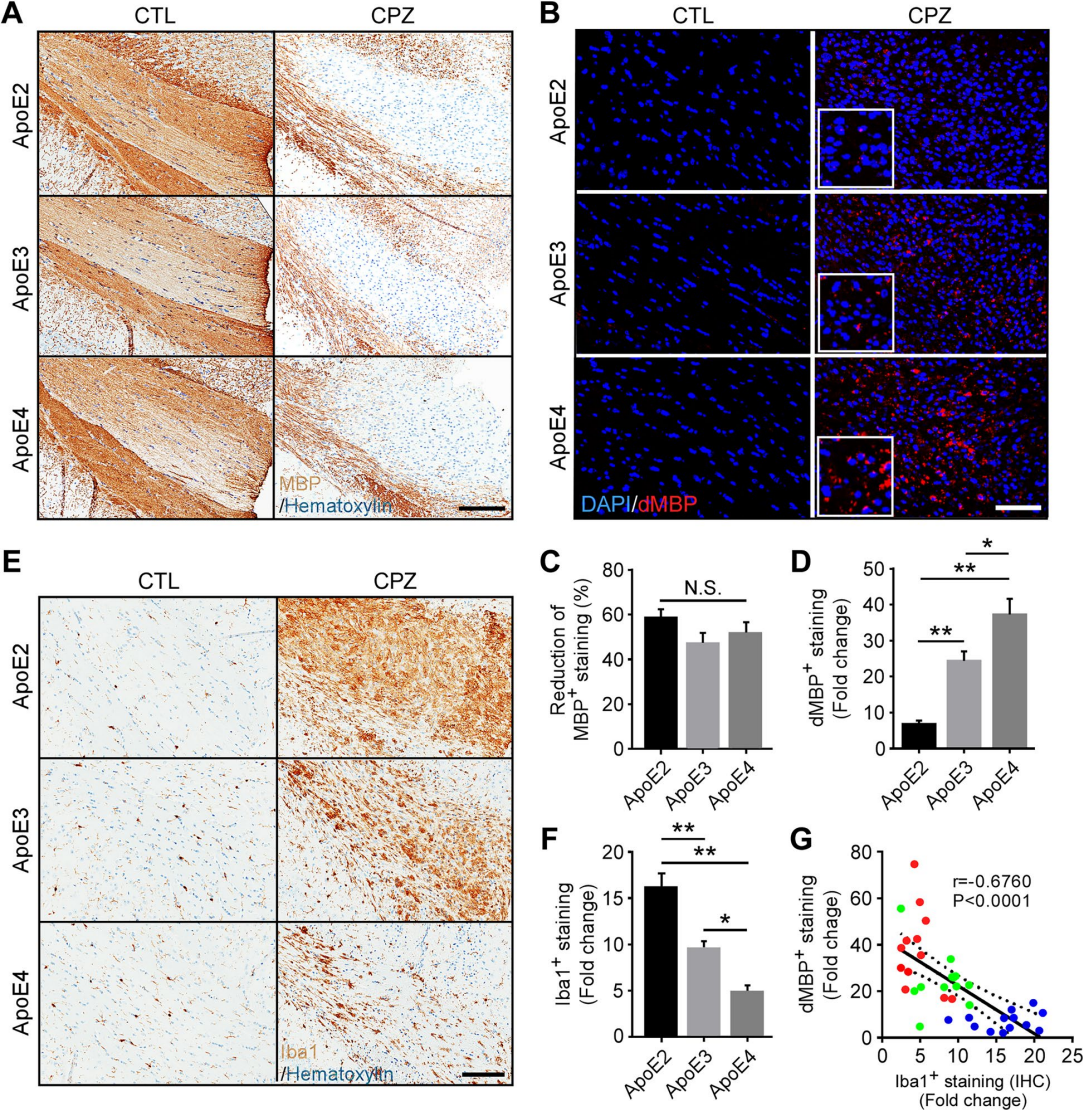
Opposing effects of apoE2 and apoE4 on microglia-mediated clearance of myelin debris in the corpus callosum of apoE-TR mice upon cuprizone-induced demyelination. ApoE-TR mice were fed with either normal diet (CTL group, *n* = 5/genotype), or cuprizone-containing diet (CPZ group, *n* = 12–13/genotype) for 4 weeks. **A** Myelin integrity in the corpus callosum (CC) of experimental mice was evaluated by immunostaining using an antibody specific to intact myelin basic protein (MBP). Representative images of the CC region are shown. Scale bar, 100 µm. **B** Myelin accumulation in the form of myelin debris was examined by immunostaining using an antibody specific to degraded myelin basic protein (dMBP). Representative images for dMBP staining are shown. Red, dMBP; Blue, DAPI. Scale bar, 50 µm. **C** The percentage (%) reduction (CPZ vs. CTL) of immunoreactivity of MBP (% Area) in the CC region was quantified. In D, F, G, the fold change represents the immunoreactivity of CPZ-treated mice compared to their respective control corresponding to their apoE genotype. **D** The immunoreactivity of dMBP (% Area) in the CC region was examined by immunofluorescence (IF) staining and the fold change (CPZ vs. CTL) was quantified. **E, F** The microglia in the CC of control and CPZ-treated apoE-TR mice were examined by immunohistochemistry (IHC) for Iba1. Representative images of Iba1 immunoreactivity are shown. Scale bar, 100 µm. The fold change (CPZ vs. CTL) of Iba1^+^ staining (%Area) was quantified. **G** A negative correlation was found between the fold change of Iba1^+^ microglia (IHC) and the fold change of dMBP^+^ myelin debris upon CPZ treatment. The Pearson correlation coefficient (r) and *P* values are shown. Blue dots, apoE2; Green dots, apoE3; Red dots, apoE4. Values are mean ± Standard error of the mean (SEM). One-way ANOVA. *P < 0.05; **P < 0.01. N.S., no significant

Microglia are the leading cell type responsible for the clearance of myelin debris upon CPZ-induced demyelination [10, 11]. To investigate the impact of apoE isoforms on microglial activation and function in response to neuronal injury, we utilized the toxin-induced demyelination model through CPZ treatment. The apoE2-TR, apoE3-TR, and apoE4-TR mice were fed with regular chow (control, CTL) or chow supplemented with 0.2% CPZ for four weeks, an established period of treatment required to induce severe CNS demyelination [10]. The CPZ treatment extensively activates microglia and elicits reactive gliosis without causing blood–brain barrier (BBB) damage [10, 57], allowing us to examine the impacts of apoE isoforms on microglial behavior upon demyelination. We first assessed the effect of CPZ treatment on brain myelin integrity in control and CPZ-treated mice by immunohistochemical staining using an antibody specific to intact myelin basic protein (MBP). Consistent with previous studies, after four weeks of treatment we observed a dramatic reduction in MBP immunoreactivity in the corpus callosum (CC), a brain region sensitive to demyelination in CPZ-treated mice compared to controls (Fig. 1A). In control mice fed with regular chow, the CC region was myelinated to the same extent across all apoE genotypes. Furthermore, no significant isoform-dependent differences in the degree of demyelination, demonstrated by reductions in MBP staining of the CC region, were observed among CPZ-treated apoE-TR mice (Fig. 1C). This indicates that a similar degree of demyelination was induced among apoE-TR mice independent of apoE isoforms.

To assess how apoE isoforms affect the clearance of damaged myelin, brain sections were immunostained using an antibody specific to degraded myelin basic protein (dMBP), which exclusively binds the MBP epitope accessible only in injured white matter areas of brain [58]. As expected, dMBP was undetectable in control apoE-TR mice (Fig. 1B). Interestingly, we found that only a limited amount of dMBP staining was detected in the demyelinated areas of the CC region in apoE2-TR mice, suggesting that myelin debris had been efficiently cleared (Fig. 1B). In contrast, dMBP immunoreactivity was moderately higher in apoE3-TR mice than the apoE2-TR group, whereas extensive accumulation of dMBP was observed in apoE4-TR mice (Fig. 1, B and D). These results indicate that apoE regulates the clearance of myelin debris upon demyelination in an isoform-dependent manner.

### Microgliosis is dramatically increased in apoE2-TR mice opposed to a limited response in apoE4-TR mice upon acute demyelination

To evaluate microglial responses upon CPZ challenge, we examined the levels of a microglial marker, ionized calcium-binding adapter molecule 1 (Iba1), in apoE-TR mice by immunohistochemical (IHC) analysis and Western blotting. Importantly, we observed apoE isoform-dependent increases of Iba1^+^ microglia in CPZ-treated mice. The Iba1^+^ immunoreactivity was dramatically increased (approximately 16-fold) in apoE2-TR mice upon CPZ treatment, whereas a relatively mild increase (approximately five-fold) was observed in apoE4-TR group (Fig. 1, E and F). Consistently, Iba1 protein levels were more abundant in apoE2-TR mice compared to those in apoE3-TR and apoE4-TR mice (Fig. sup 1, A and B). Intriguingly, the amount of microglia signal was negatively correlated with the amount of myelin debris in apoE-TR mice (Fig. 1, G and Fig. sup 1, C). This result indicates that apoE2 microglia are highly responsive to acute demyelination, whereas apoE4 microglia fail to respond efficiently to neuronal injury upon CPZ-induced demyelination.

### ApoE isoforms differentially regulate proliferation and morphological changes of microglia in apoE-TR mice upon cuprizone-induced demyelination

**Fig. 2:**
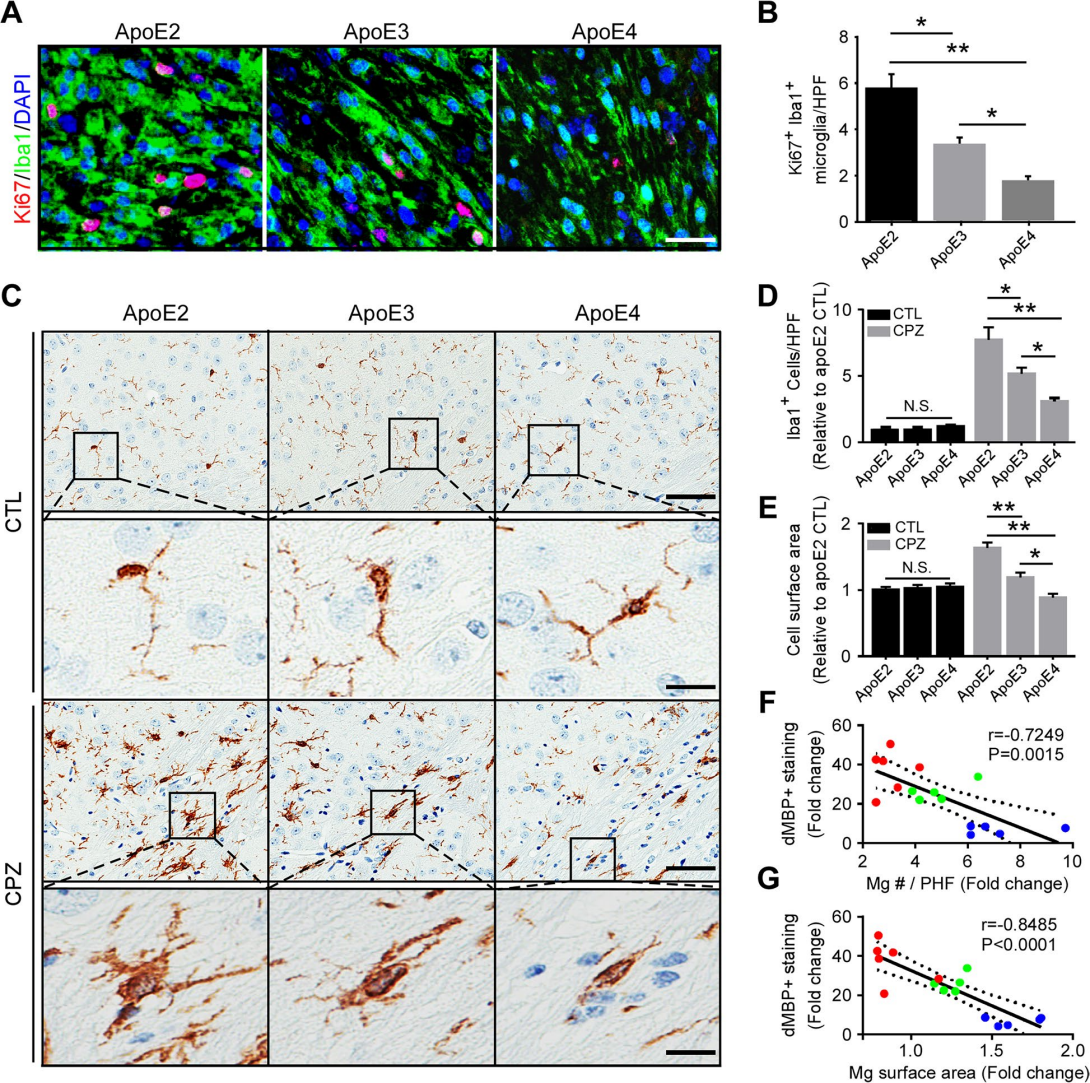
ApoE isoform-dependent effects on microglial proliferation and morphological changes. **A** Brain samples from CPZ-treated apoE-TR mice (*n* = 12–13/genotype) were subjected to immunofluorescence staining with Ki67 (for proliferation) and Iba1 (for microglia). Representative images of Ki67^+^Iba1^+^ microglia upon CPZ-induced demyelination are shown. Scale bar, 15 µm. **B** The number of Ki67^+^Iba1^+^ microglia per high-power field (HPF) was quantified. **C** Representative images of Iba1^+^ microglia in the CC area are shown. Scale bar, 25 µm (low magnification); 10 µm (high magnification). **D** The numbers of Iba1^+^ microglia per high-power field (HPF; 3 HPFs/mouse) were analyzed. **E** The surface area of Iba1^+^ microglia in the CC area were quantified. **F** A negative correlation was observed between the fold change of microglia number (Mg #) and the fold change of dMBP^+^ staining upon CPZ treatment. **G** A negative correlation was shown between the fold change of microglial surface area and the fold change of dMBP^+^ staining upon CPZ treatment. Blue dots, apoE2; Green dots, apoE3; Red dots, apoE4. The Pearson correlation coefficient (r) and P values are shown. Values are mean ± SEM. Two-way ANOVA. *P < 0.05; **P < 0.01. N.S., not significant

As microglia dramatically increased upon CPZ treatment, we next examined the proliferation of resident microglia by co-immunostaining of Ki67, a proliferation marker, and Iba1 (Fig. 2A). The number of proliferating microglia was significantly higher in apoE2-TR mice compared to those in apoE3-TR and apoE4-TR mice (Fig. 2B), indicating that apoE regulates microglial proliferation in an isoform-dependent manner upon demyelination. Considering the possibility of peripheral immune cells infiltrating through a compromised blood–brain barrier (BBB), we examined the BBB integrity by analyzing the tight junction protein claudin-5 (CLDN5) in endothelial cells. We found that the levels of CLDN5 normalized by vascular marker Glut1 were similar among apoE-TR mice (Fig. sup 2, A and B), indicating no differences in BBB integrity of apoE-TR mice upon CPZ-induced demyelination. These findings indicate that resident microglia play a major role in myelin debris clearance in responses to CPZ challenge.

Upon injury, microglia are activated and markedly change their morphology by enlarging their soma and retracting their extensions to form processes that are short and stubby in appearance [59–61]. Abnormal morphological changes of microglia in response to CNS injury are thought to reflect the senescent phase of degenerating microglia as found in aged human brains and those following traumatic brain injury [62, 63]. Therefore, we investigated whether apoE isoforms have differential impacts on the morphological changes of reactive microglia upon acute demyelination. The number of microglial cells was quantified under high-power field (HPF). CPZ treatment increased the number of Iba1^+^ cells, which is consistent with the results in Fig. 1. Importantly, the number of microglia was dramatically increased (eight-fold) in apoE2-TR mice, whereas there was only a moderate increase (three-fold) in the number of microglia observed in apoE4-TR mice upon demyelination (Fig. 2, C and D). Furthermore, we found that apoE2 microglia had larger cell bodies with more extensive ramifications and had become hyperactive in response to demyelination compared to apoE3 microglia (Fig. 2, C and E). In contrast, Iba1^+^ microglia in the CPZ-treated apoE4 group were less activated, as indicated by smaller somas and fewer ramifications (Fig. 2, C and E). Moreover, compared to those in control animals, the fold increase in the quantity as well as cell surface area of microglia upon CPZ challenge were negatively correlated with myelin debris accumulation according to apoE genotype (Fig. 2, F and G). These morphological differences demonstrate that apoE2 microglia are highly responsive to acute demyelination, whereas such responses are defective in apoE4 microglia.

Given that astrogliosis accompanies microgliosis in CPZ-induced demyelination [57], we determined whether astrocytic morphological changes occur in the CC region of apoE-TR mice treated with CPZ. Immunostaining of glial fibrillary acidic protein (GFAP)-positive astrocytes was increased by equal amounts upon demyelination among apoE-TR mice with different isoforms (Fig. sup 3, A and C). In addition, compared to control mice, the quantities and degree of morphological change of astrocytes were similar among CPZ-treated apoE-TR mice (Fig. sup 3, B, D and E). Moreover, a set of selected astrocyte-enriched genes [64] revealed similar changes in the transcriptomic signature following CPZ-induced injury for each apoE genotype (Fig. sup 4). These results indicate that differential clearance of myelin debris among apoE-TR mice following acute demyelination is likely attributed to microgliosis, but not astrogliosis.

### Pathways associated with microglial functions are robustly altered in apoE2-TR mice, while minimally changed in apoE4-TR mice upon acute demyelination

**Fig. 3:**
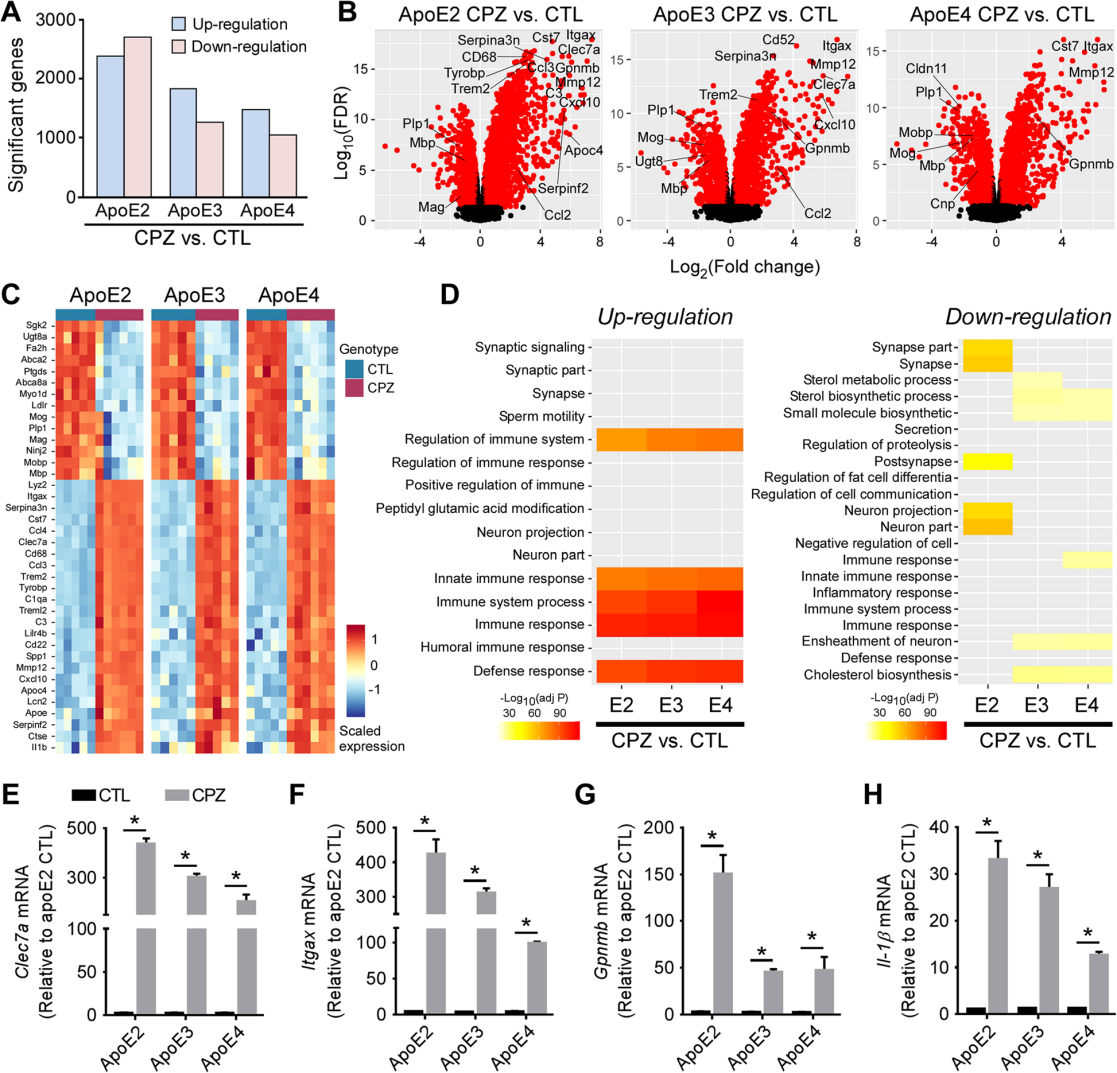
The transcriptomic profiles of apoE-TR mice upon cuprizone-induced demyelination. RNA extracted from the CC area of control (*n* = 5/genotype) and CPZ-treated (*n* = 5–6/genotype) apoE-TR mice was subjected to transcriptomic profiling. **A** An overview of up-regulated and down-regulated gene changes in apoE-TR mice upon CPZ treatment. **B** Volcano plot representation of differentially expressed genes (DEGs, CPZ vs. CTL for each apoE isoform). Genes significant at the FDR adjusted *P* value ≤ 0.05 and fold change ≥ 1.2 are denoted red in color. The X-axis represents log2 transformed fold-change (CPZ vs. CTL) and y-axis represents the -log10 adjusted *P* value. **C** Heat map representing transcriptional expression of selected genes in the CPZ and CTL groups. The scaled expression value (row Z score) is shown with a blue-red color scheme, denoting red as higher expression and blue as lower expression. **D** The up-regulated and down-regulated pathways in apoE-TR mice upon CPZ treatment compared to control animals. **E–H** The expression of immune response-associated genes (e.g., *Clec7a, Itgax, Gpnmb,* and *Il-1β*) was validated by real-time PCR. Values are mean ± SEM. Mann–Whitney tests followed by Bonferroni correction for multiple comparisons were used. *P < 0.0167

As microglial behaviors differed considerably among apoE-TR mice upon demyelination, we next investigated the molecular mechanisms by which apoE isoforms modulate microglial functions in response to CPZ. We performed transcriptomic profiling on the CC region of apoE-TR mice with or without CPZ treatment. We first examined the differentially expressed genes (DEGs) between control and CPZ-treated mice with respect to *APOE* genotype. We found 1,000 to 2,800 DEGs (FDR-adjusted P value ≤ 0.05 and fold change ≥ 1.2) up-regulated or down-regulated between control and CPZ-treated apoE-TR mice with apoE2-TR mice displaying the highest numbers of DEGs (Fig. 3A). A selected set of up and down-regulated genes revealed a clear visual separation between the control and CPZ-treated groups and for each apoE genotype, reflecting distinct changes in the transcriptomic signature following CPZ-induced injury. As expected, several genes involved in the regulation of the myelination process or myelin structures (e.g., *Plp*, *Mbp*, *Mobp*, *Ugt8*, *Cldn11*, *Cnp*, and *Mog*) were significantly down-regulated (Fig. 3, B and C). In addition, chemokines, complement factors, and numerous genes related to microglial activation, as well as DAM genes (e.g., *Csf1*, *Csf1r*, *Ccl2*, *Ccl3*, *Ccl4*, *C3*, *C1qa*, *Itgax*, *Gpnmb*, *Serpina3n*, *Clec7a*, *Spp1*, *Trem2* and *Cst7*) were dramatically up-regulated in CPZ-treated apoE-TR mice compared to control mice (Fig. 3, B and C). Pathway analyses of DEGs revealed that the top up-regulated pathways were enriched in defense and immune responses in all apoE-TR mice upon CPZ challenge (Fig. 3D). Sterol metabolism and cholesterol biosynthesis were down-regulated in apoE3-TR mice and apoE4-TR mice, whereas neuron projection was down-regulated in apoE2-TR (Fig. 3D). To validate the transcriptomic changes, the expression of key genes of interest were examined by quantitative real-time PCR. DAM genes *Clec7a*, *Itgax* and *Cst7* are associated with innate immune responses in the setting of neurological diseases [65–67]. Monocyte chemoattractant protein (*Ccl2/Mcp-1*), colony-stimulating factor 1 (*Csf1*) and its specific receptor (*Csf1r*) play crucial roles in inflammatory response, microglial activation, and proliferation [68–71]. We showed that the expression of these genes was markedly increased in CPZ-treated apoE-TR mice compared to control mice (Fig. 3, E and F; Fig. sup 5, A-D), validating the findings in transcriptomic profiling. Similarly, genes associated with DAM and neuroinflammation (e.g., *Gpnmb*, *Serpina3n, Tnf-α* and *Il-1β*) were dramatically increased upon demyelination (Fig. 3, G and H; Fig. sup 5, E and F).

**Fig. 4:**
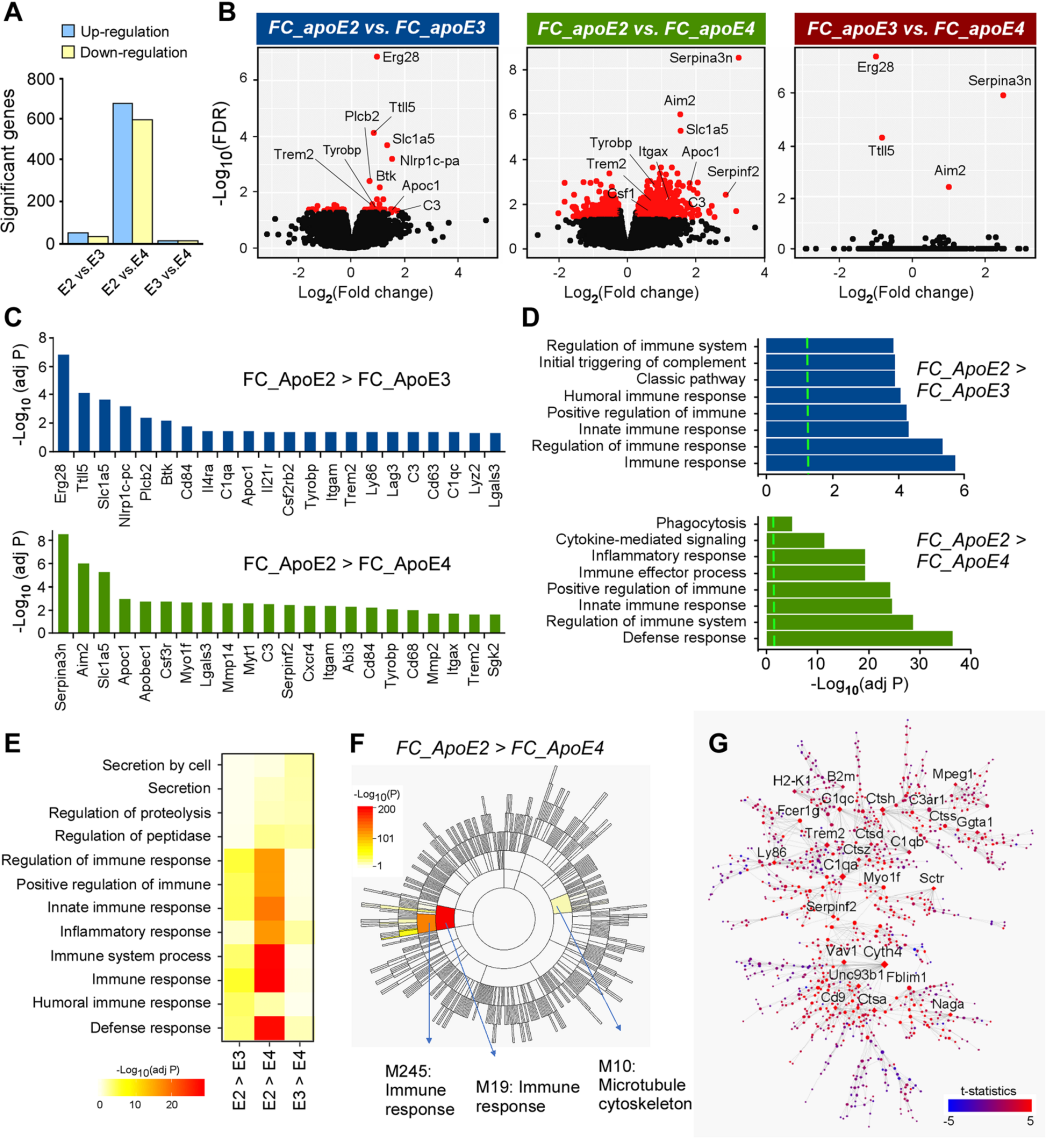
ApoE isoform-dependent transcriptional changes in mice upon cuprizone-induced demyelination. **A** An overview of genes with significant difference in fold change (FC) from CPZ *vs* control groups, among three apoE isoforms. **B** Volcano plot representation of gene FC among different apoE-TR mice treated with CPZ. The representative DEGs are shown. X-axis represents log2 fold change (CPZ vs CTL) and y-axis represents -log10 FDR adjusted P value. **C** Chart showing selected genes with significant fold changes identified through transcriptomics after CPZ treatment (E2 > E3 and E2 > E4). **D** Critical pathways enriched for the genes with significant FC difference. **E** Heat map showing main differentially regulated functional pathways. **F** Sunburst plot showing the MEGENA co-expression module hierarchy. Modules enriched for genes with significantly increases in FC in apoE2 compared to apoE4 are labeled. **G** Network topology of immune response module M245 is illustrated using Cytoscape. Node size is proportional to the network degree such that genes that harbor more relationships with other molecules have greater size. Labels are shown for nodes connected to at least 16 other nodes. Node color intensity denotes the differential expression t-statistics of comparing fold change between apoE2 and apoE4. Diamond shape denotes hub gene

To explore the molecular networks that are differentially regulated by apoE genotype upon CPZ treatment, we examined the gene fold-changes (FC) of CPZ-treated versus control apoE-TR mice. The fold change of genes in apoE2-TR mice (FC_ApoE2 represents CPZ-induced changes vs. control treatment in apoE2 mice) were compared to those in apoE3-TR (FC_ApoE3) or apoE4-TR (FC_ApoE4) mice. Interestingly, the highest numbers of DEGs were identified when the gene fold-change in apoE2-TR mice (FC_ApoE2) were compared with those in apoE4-TR mice (FC_ApoE4) after CPZ treatment (Fig. 4A). We found that numerous important genes related to microglial functions (e.g., *Trem2, Tyrobp, Mmp2, Cd68, Apoc1* and *Btk*) were highly up-regulated in apoE2-TR mice compared with apoE3-TR or apoE4-TR mice upon demyelination (Fig. 4, B and C). Gene Ontology (GO) enrichment analysis revealed the most prominent changes pertained to the immune and inflammatory responses, phagocytosis, and defense mechanisms when comparing gene fold-change in apoE2-TR mice to apoE3-TR or apoE4-TR mice upon CPZ treatment (Fig. 4, D and E). Furthermore, as the largest phenotypic difference was observed between apoE2-TR and apoE4-TR mice, we employed multiscale embedded gene co-expression network analysis (MEGENA) [56] to explore the networks that contribute to this phenotype. Upon demyelination, apoE2-TR mice exhibited a drastic increase of immune response and microtubule cytoskeleton pathways associated with cell migration and phagocytosis compared to apoE4-TR mice [72] (Fig. 4F). This immune response module was characterized by an enrichment of phagosomal (*B2m*) and lysosomal functions (*Ctsd, Ctss, Ctsh, Ctsz*), migration/adhesion pathways (*Myo1f, Fblim1*), membrane trafficking (*Cyth4*), complement components (*C1qa, C1qb*), and lipid homeostasis (*Trem2*) (Fig. 4G). Taken together, these results suggest that apoE2 microglia exhibit an overall stronger inflammatory response as well as greater migratory and phagocytic abilities compared to apoE4 microglia in response to neuronal injury.

We further validated the expression of genes known to be essential in regulating microglial functions. Specifically, *Trem2, Tyrobp, Aim2, Btk, B2m, Ctsd*, *C3* and *CR3* play important roles in microglial activation, inflammation, and phagocytosis [49, 73–76]. In addition, *Mmp2* and *Mmp12* regulate microglial migration to the injury sites, a prerequisite for subsequent inflammatory responses [3]. Clearance of myelin debris is mediated by activated microglia, which display an up-regulation of phagocytic markers (*Cd68* and *Axl*) [73, 77]. Using real-time PCR, we confirmed that these genes were significantly up-regulated in apoE-TR mice in an isoform-dependent manner, where the highest expression was observed in apoE2-TR mice, and the lowest found in apoE4-TR mice (Fig. sup 6). The apoE isoform-dependent changes of these important genes and related pathways are associated with the differential functions of microglia upon myelin damage. Collectively, our results demonstrate that apoE2 promotes microglial activation, immune response, cellular migration, phagocytosis, and lipid metabolism in response to neuronal injury, whereas apoE4 compromises these microglial functions.

### Reduced phagocytic ability of apoE4 microglia upon demyelination

**Fig. 5:**
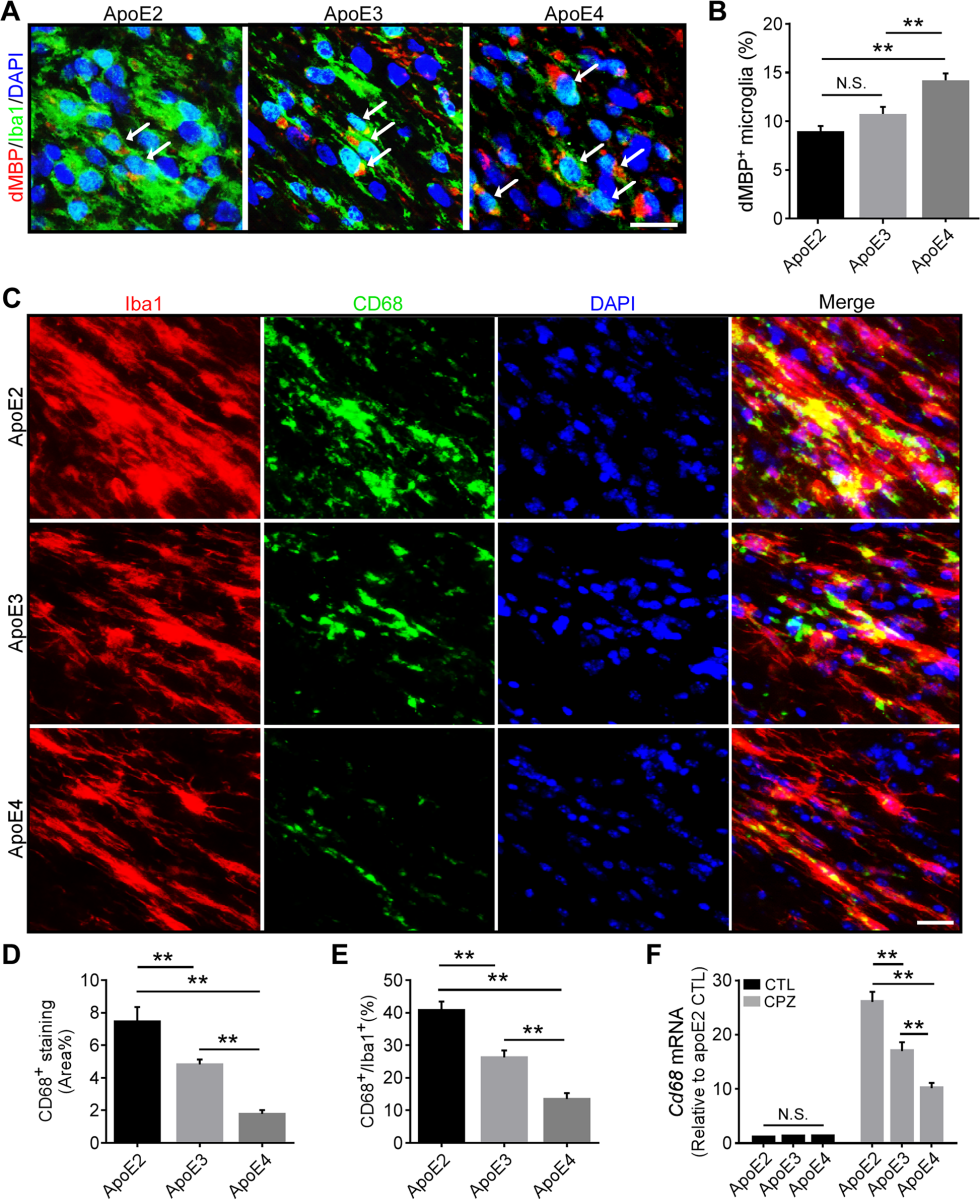
Opposing effects of apoE2 and apoE4 on microglial phagocytic ability upon cuprizone-induced demyelination. **A** Brain samples from CPZ-treated apoE-TR mice (*n* = 12–13/genotype) were subjected to immunofluorescence staining for dMBP (for myelin debris) and Iba1 (for microglia). Representative images of dMBP^+^ (Red) and Iba1^+^ (Green) microglia in the CC region of apoE-TR mice treated with CPZ. Blue, DAPI. Scale bar, 15 µm. **B** The percentage of dMBP-accumulated microglia (pointed by arrow) in the CC of experimental mice was quantified. **C** Brain samples from CPZ-treated apoE-TR mice (*n* = 16/genotype) were subjected to immunofluorescence staining for CD68 (for microglial phagocytosis) and Iba1 (for microglia). Representative images of CD68^+^ (Green) and Iba1^+^ (Red) microglia in the CC region of apoE-TR mice treated with CPZ. Blue, DAPI. Scale bar, 35 µm. **D** The percentage of CD68^+^ area in the CC of experimental mice was quantified. Values are mean ± SEM. One-way ANOVA. * *P* < 0.05; ** *P* < 0.01. **E** The ratio of CD68 signal normalized to Iba1^+^ microglial signal was quantified. **F** The expression of *cd68* was examined by real-time PCR (*n* = 5–6 mice per group). Values are mean ± SEM. Two-way ANOVA. ** *P* < 0.01. N.S., not significant

Microglia play a critical role in the clearance of myelin debris after acute demyelination in CNS [26]. ApoE4 microglia may have reduced ability to uptake and/or to degrade myelin fragments, leading to the accumulation of myelin debris observed in apoE4-TR mice (Fig. 1B). Using co-immunostaining for dMBP (for myelin debris) and Iba1, we found that myelin debris accumulates inside microglia in an apoE isoform-dependent manner with the most abundant debris observed in apoE4 microglia and least in apoE2 microglia (Fig. 5, A and B). We further analyzed the phagocytic function of microglia in apoE-TR mice by staining with antibodies against CD68 (a marker for phagocytic microglia) and Iba1. Intriguingly, CD68^+^ area and the levels of CD68 normalized by Iba1 were both significantly reduced in apoE4-TR mice (Fig. 5, C-E). Consistently, the expression of *Cd68* was lower in apoE4-TR and higher in apoE2-TR mice compared to that in apoE3-TR mice (Fig. 5 F). These findings indicate that apoE4 microglia exhibit reduced phagocytic function and degradation ability, leading to the accumulation of myelin debris in apoE4-TR mice.

### Enhanced lipid droplet accumulation in apoE4 microglia upon demyelination

**Fig. 6:**
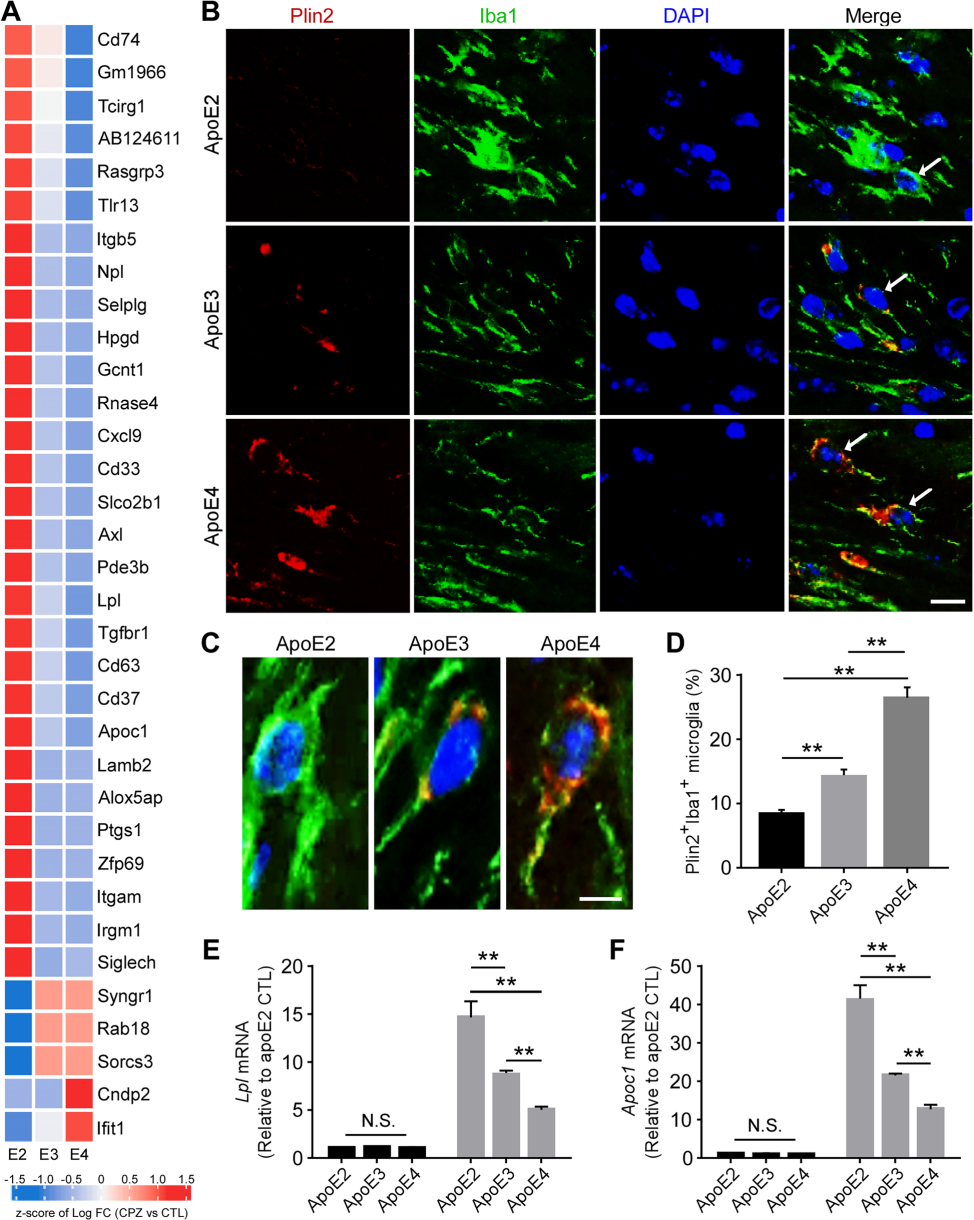
Opposing effects of apoE2 and apoE4 on lipid droplet accumulation in the microglia of apoE-TR mice upon cuprizone-induced demyelination (**A** Heat map showing transcriptional changes (CPZ vs CTL groups) of selected genes associated with lipid metabolism identified from RNA-Sequencing. The scaled expression value (row Z score) is shown with a blue-red color scheme, denoting red as higher expression, and blue as lower expression. **B** Brain samples from CPZ-treated apoE-TR mice (*n* = 12–13/genotype) were subjected to immunofluorescence staining for Plin2 (for lipid droplets) and Iba1 (for microglia). Representative images of Plin2^+^ (Red) Iba1^+^ (Green) microglia in the CC region of apoE-TR mice treated with CPZ. Blue, DAPI. Scale bar, 15 µm. **C** Zoomed-in images for accumulated Plin2^+^ lipid droplets in microglia with different apoE genotypes. Scale bar, 10 µm. **D** The percentage of Plin2^+^ and Iba1^+^ microglia in the CC region of experimental mice were quantified. Values are mean ± SEM. One-way ANOVA. ** *P* < 0.01. **E–F** The expression of genes involved in microglial lipid metabolism (*Lpl* and *Apoc1*) was examined by real-time PCR. *n* = 5–6 mice per group. Values are mean ± SEM. Two-way ANOVA. ** *P* < 0.01. N.S., not significant

The clearance of myelin-derived lipids by microglia is required for optimal remyelination following demyelination [13, 78]. With aging and the development of neurodegenerative diseases, microglia have been shown to lose their ability to effectively efflux cholesterol, leading to an accumulation of cholesterol-rich myelin debris in lipid droplets (LDs) [24, 79]. Several studies show that lipid droplet accumulating microglia (LDAM) exhibit deficits in phagocytic capacity and produce excessive amounts of proinflammatory cytokines and reactive oxygen species [16, 18, 80], and are implicated in the pathogenesis of neuroinflammatory diseases. Interestingly, transcriptomic analysis revealed an apoE isoform-dependent modulation in genes associated with lipid metabolism and LD formation [18, 79, 81] (Fig. 6A).

To examine whether apoE isoforms affect lipid accumulation in microglia, we investigated the accumulation of LDs in microglia by co-immunostaining for Iba1 and perilipin 2 (Plin2), one of the most highly expressed LD-associated proteins (Fig. 6 B and C). Importantly, we found that the percentage of Plin2^+^ microglia was much higher in apoE4 mice (26%) compared to apoE2 (9%) and apoE3 (13%) mice (Fig. 6D). We next validated genes associated with lipid transport and metabolism and showed that *Lpl* and *Apoc1* were altered in an apoE isoform-dependent manner. Interestingly, we observed the highest expression levels of *Lpl* and *Apoc1* in apoE2 mice and lowest expression levels in apoE4 mice (Fig. 6, E and F). Together, these results indicate that apoE isoforms differentially regulate lipid metabolism, which impacts microglial functions in the clearance of myelin debris upon neuronal injury.

### Enhanced remyelination in apoE2-TR mice after cuprizone removal

**Fig. 7:**
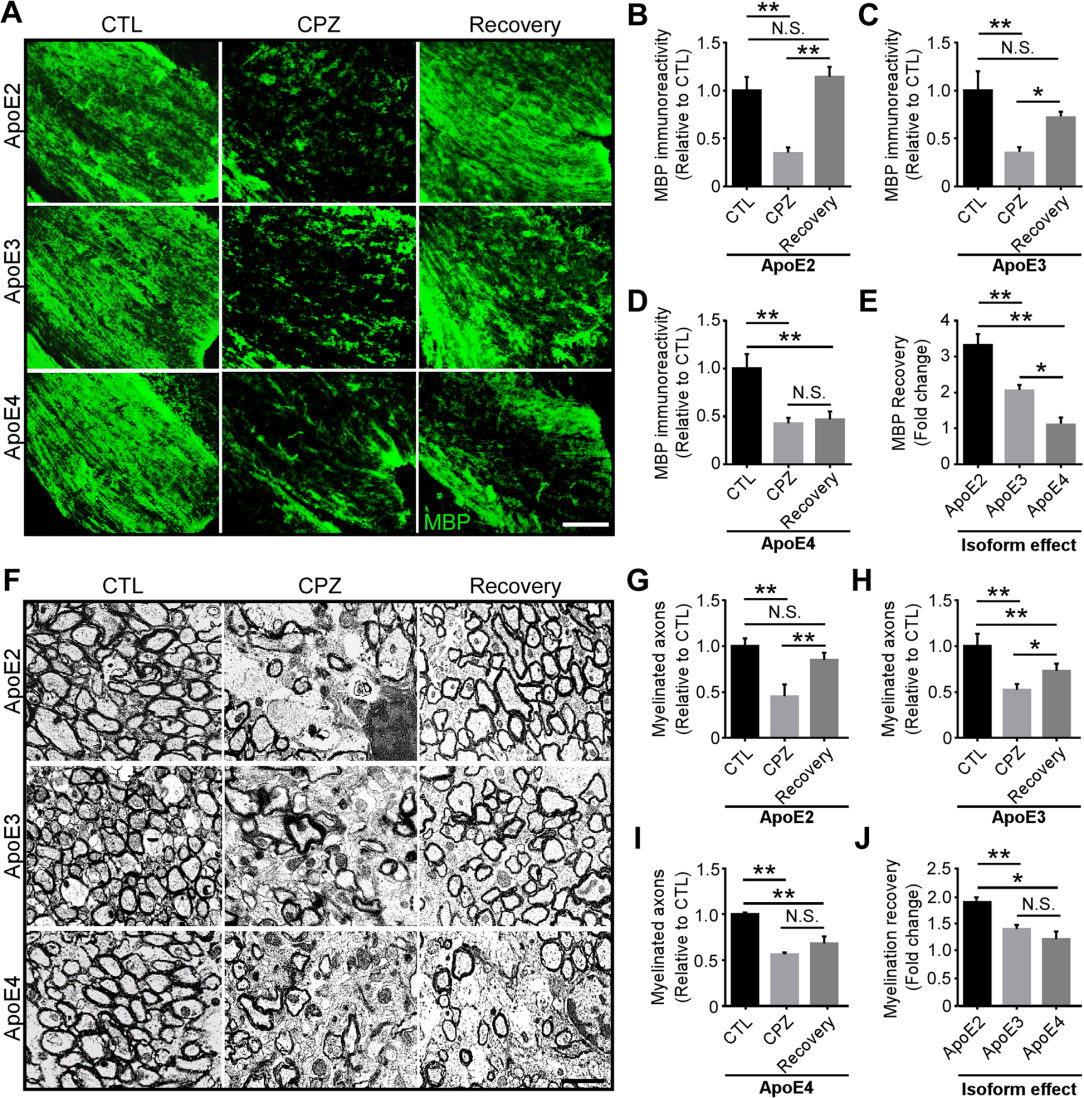
Differential effects of apoE isoforms on remyelination after cuprizone removal. ApoE-TR mice were fed with either normal diet (CTL) or CPZ diet for four weeks, following which CPZ diet was switched back to normal chow for two weeks to allow myelin recovery. **A** Brain samples of apoE-TR mice from different treatment groups (CTL, *n* = 9; CPZ and Recovery, *n* = 14–16/genotype) were subjected to immunofluorescence staining for MBP. Representative images of MBP^+^ (Green) staining in the CC region of apoE-TR mice are shown. Scale bar, 50 µm. **B**-**D** The immunoactivity of MBP was quantified. **E** MBP recovery (fold change of Recovery vs. CPZ) in CC area of apoE-TR mice was quantified. **F** Brain samples of apoE-TR mice from different groups (CTL, CPZ and Recovery, *n* = 4/genotype) were subjected to transmission electron microcopy (TEM) analysis. Representative images of myelinated axons in the CC region of apoE-TR mice are shown. Scale bar, 50 µm. **G**-**I**, Myelinated axons in the CC region of apoE-TR mice were quantified. **J** The extent of remyelination (fold change of Recovery vs. CPZ) in different apoE-TR mice was quantified. Values are mean ± SEM. One-way ANOVA. * *P* < 0.05; ** *P* < 0.01. N.S., not significant

The microglial activation and efficient clearance of myelin debris are critical for subsequent remyelination after CPZ removal [82]. Given that apoE isoforms differentially affect microglial functions and their clearance of myelin debris, we further explored their effects on myelin recovery. To determine the extent of remyelination in apoE-TR mice, we replaced CPZ diet with normal diet for an additional two weeks after four weeks of CPZ treatment. Importantly, an efficient myelin recovery throughout the CC region was observed in apoE2-TR mice examined by immunostaining and transmission electron microscopy (TEM) analysis (Fig. 7, A, B, F and G). Also, significant increases of MBP^+^ myelin and myelinated axons were observed in apoE3-TR mice compared with CPZ group (Fig. 7, A, C, F, H), whereas minimal myelin recovery was observed in apoE4-TR mice (Fig. 7, A, D, F, I). In addition, apoE regulates the amount of remyelination in an isoform-dependent manner, with the most efficient myelin recovery observed in apoE2-TR mice compared to apoE3-TR and apoE4-TR mice (Fig. 7, E and J). All together, these results suggest that efficient clearance of myelin debris in apoE2-TR mice enhances remyelination, whereas microglial dysfunction may contribute to the compromised myelin recovery in apoE4-TR mice.

## Discussion

Emerging large-scale genetic studies and transcriptomic profiling have determined that microglia play pivotal roles in brain homeostasis, and may serve as a potential therapeutic target for neurodegenerative diseases [41, 74, 83–87]. Upon myelin damage, microglia can remove myelin debris and accumulated lipids from the brain, which is crucial for subsequent tissue repair [27, 28]. ApoE has been shown to modulate microglial function and lipid transport [32, 33, 41, 88]; however, the differential effects of apoE isoforms in regulating microglial behavior in response to myelin damage in vivo remain unclear. In this study, we demonstrated that apoE impacts microglial responses to acute demyelination in an isoform-dependent manner with microglia becoming hyperactive in apoE2 mice but less activated in apoE4 mice. Immunohistochemical analysis and transcriptional profiling indicated that apoE isoforms differentially regulate the activation of microglia, proliferation, morphological changes, as well as the expression of inflammation-related genes. These findings suggest that apoE isoforms affect microglial response in an isoform-dependent manner, which may in turn influence the clearance of myelin debris and associated lipids. Intriguingly, apoE4 microglia exhibit an increase of myelin debris and lipid droplet accumulation which may further compromise microglial functions. Indeed, poor remyelination was also observed in apoE4-TR mice, whereas apoE2 is associated with the most efficient remyelination. Collectively, our study highlights a key role of apoE in microglial activation in response to CNS injury such as acute demyelination. As such, targeting apoE-dependent microglial functions may present a new therapeutic avenue for the treatment of neuronal disorders.

Dysregulation of lipid metabolism and lipid droplet accumulation in microglia has been associated with aging and neurological disorders [8, 18, 41]. Importantly, lipid metabolism is reported to modulate microglial activation and essential functions such as phagocytosis, migration, and immune response [17, 18, 89]. ApoE is known to mediate brain lipid transport and recycling, which is crucial for the production, maintenance, and repair of myelin [90–92]. Our results show an opposing effect of apoE2 and apoE4 on myelin debris and LD accumulation in microglia which may contribute to their differential clearing efficiencies of myelin debris upon myelin damage. Thus, reducing apoE-mediated myelin debris and LD accumulation may be beneficial in maintaining microglial homeostasis to mitigate neurodegenerative disease.

ApoE has also been shown to be a pleotropic modulator of neuroinflammatory responses [93–95]. Moreover, apoE mediates the microglial transition from a homeostatic to a neurodegenerative phenotype [41]. The expression of *Apoe* is highly induced when microglia are activated, suggesting that apoE may regulate microglial activation and function through their specialized immunomodulatory sensitivity [9, 36]. As such, it is important to investigate how apoE isoforms affect microglial behaviors in the context of neuronal disorders or other CNS injuries. Using a CPZ-induced demyelination model, we demonstrated that apoE regulates microglial activation in an isoform-dependent fashion. Compared to apoE3 microglia, apoE2 microglia were highly activated and more readily responsive to neuronal injury, whereas the responsiveness of apoE4 microglia was compromised. We also showed that markers related to microglial activation, such as *Csf1r*, *Tyrobp,* and *Itgax*, were changed differentially according to *APOE* genotype. The activation status of microglia affects the efficiency of their subsequent responses including migration and neuroinflammation [4, 96–99]. Consistent with this notion, we showed that migration (*Mmp2*, *Mmp12*) and inflammation-related markers (*Tnf-α*, *Il-1β*) were up-regulated in an apoE isoform-dependent manner, with a higher level in the CC region of apoE2-TR mice and a lower level in apoE4-TR mice. These results suggest that microglia with different apoE isoforms exhibit differential activation states and immunomodulatory functions, which may account for their beneficial or detrimental roles in the pathogenesis of neurodegenerative diseases. In addition, it is suggested that acute microglial activation is a protective response which aims to limit the deleterious effects of neuronal damage, whereas chronic microglial activation might represent a more detrimental state with a prolonged pro-inflammatory response, potentially causing damage to healthy brain cells [100]. The elevated activation and responsiveness of apoE2 microglia might contribute to their protective functions in the early stages of neuronal injury, whereas a prolonged activation status of microglia may lead to detrimental effects in the later stages after injury or during chronic neurological diseases. Microglia expressing apoE4 may lose their protective role in the initial stage of CNS injury, resulting in reduced overall responses to neuronal damage and an inefficient clearance of myelin debris.

The phagocytic clearance of microglia plays a fundamental role in monitoring neuronal homeostasis and triggering tissue repair [26, 101]. Increasing evidence suggests that alterations in myelination integrity are associated with neurodegenerative diseases, where myelin debris clearance is an essential action for subsequent repair [90, 102, 103]. Our study showed that myelin debris is efficiently cleared in apoE2-TR mice, whereas this clearance is impaired in apoE4-TR mice. As myelin debris are cholesterol-rich, it is suggested that defective cholesterol clearance results in accumulation of myelin debris, which limits cholesterol synthesis and subsequent remyelination [15]. The down-regulated cholesterol synthesis pathways in apoE4 mice may lead to insufficient clearance of damaged myelin by microglia with excessive accumulation of cholesterol. As a result, efficient remyelination was observed in apoE2 mice, whereas myelin recovery was poor in apoE4 mice. Recent studies found that DAM cells, concentrated around Aβ plaques, exhibit up-regulated genes involved in lipid metabolism and phagocytosis, such as Cst7 and Lpl [74, 104]. Interestingly, we showed that several phagocytic and lipid transport related genes (i.e., Cd68, Cst7, Axl, Lpl and Apoc1) were increased upon demyelination in an apoE isoform-dependent manner. In addition, we found that TREM2 levels are up-regulated in an apoE isoform-dependent manner after CPZ treatment. Deficiency of TREM2 has been shown to impair microglial functions by reducing their phagocytic ability [49, 73, 105]. As a ligand of TREM2, it is possible that apoE isoforms differentially regulate microglial activities by interacting physically and functionally with the TREM2 receptor on microglia [47].

Various studies explored the effects of *APOE* genotype in AD mouse model, human brain, and iPSC-derived microglia, and showed that apoE4 was associated with proinflammation in transcriptomic analysis. Intriguingly, one of these studies showed that apoE4 microglia were unable to switch to an activated state critical for Aβ clearance, likely contributing to the increase of amyloid pathology in AD patients carrying *APOE4* [106]. In addition, in the iPSC study, though the apoE4 microglia-like cells were associated with an increase of immune response in the transcriptomic analysis, they exhibited altered morphologies, and reduced Aβ phagocytosis [107], suggesting that these microglia are functionally impaired. Also, while *APOE4* astrocytes secrete more inflammatory cytokines, *APOE4/4* iPSC-derived microglia display lipid accumulation associated with dysregulation of lipid metabolism [108]. Our study showed that apoE4 microglia exhibited deficits in proliferation, phagocytosis, myelin debris clearance in responses to CPZ-induced brain injury, whereas apoE2 microglia were highly responsive, displaying superior functions in clearing myelin debris which facilitated remyelination. Although there is seemingly discrepancy in the transcriptomic changes in inflammatory responses in these studies, apoE4 microglia have been consistently shown to exhibit functional deficits. Furthermore, the effects of apoE isoforms on microglial responses may be context-dependent and can be influenced by pathological conditions, disease stages, model systems and if they are compensatory changes, which warrant further investigation.

Mounting evidence indicates that microglia exhibit different cellular phenotypes and functions during brain development and disease progression [17, 18, 109]. Importantly, apoE, whose gene is a major genetic risk factor for AD, has been shown to regulate microglial functions in AD pathogenesis [32, 39, 41–44, 110]. We thus explored the effects of apoE isoforms on microglial responses upon CPZ-induced neuronal injury. Although AD is not generally considered or classified as a demyelinating disease, myelin impairment plays an important role in AD pathogenesis [111–113]. Numerous studies have shown early and robust transcriptional changes in myelin- and oligodendrocyte-specific genes in AD [114, 115]. Additionally, the levels of myelin-associated proteins and lipids are reduced in the early stages of AD and in mild cognitive impairment (MCI) [116, 117]. Extensive myelin loss has been observed in individuals with AD, while enhancing myelin renewal could rescue cognitive deficits in amyloid mouse models [111, 118, 119]. Furthermore, focal demyelination in AD patients and mouse models is assciated with Aβ and neurofibrillary pathology [115, 120]. Thus, understanding how apoE isoforms regulate microlgial behaviors and their impacts on demyelination and myelin recovoy may shed lights on their roles in AD and other neurodegenerative diseases.

Microgliosis may initially serve a neuroprotective role upon injury; however, prolonged microglial activation may result in over production of pro-inflammatory cytokines and reactive oxygen species. As a result, neurotoxicity and excessive loss of synaptic proteins can be observed in the context of neurodegeneration [121–123]. We observed a down-regulation of synapse-related pathways in apoE2-TR mice, likely resulting from prolonged activation of microglia. Future studies investigating the roles of microglia-specific expression of apoE at different stages of neuronal injury and in different CNS injury models would provide additional insights into the mechanisms by which apoE isoforms differentially influence the innate immune responses over the time course of the disease.

## Conclusions

In conclusion, our study demonstrates apoE isoform-dependent differences in microglial responses upon myelin damage. Compared with apoE3 microglia, apoE4 microglia exhibit deficits in microglial activation, proliferation, myelin debris clearance, and subsequent remyelination, whereas apoE2 microglia display enhanced microglial function. Overall, our findings indicate that apoE plays a critical role in modulating microglial responses and related lipid metabolism upon neuronal injury, which may contribute to the development of neurodegenerative diseases. By illustrating how apoE isoforms differentially regulate microglial function, our study sheds light on the therapeutic premise of strategies that target the microglia-mediated responses for treating neurological diseases.

## Supplementary Information

**Additional file 1: Figure sup 1.** Opposing effects of apoE2 and apoE4 on Iba1 proteinlevels and their correlation with the amount of myelin debris in the corpuscallosum of apoE-TR mice upon cuprizone-induced demyelination. ApoE-TR micewere fed with either normal diet (CTL, *n*=5/genotype), or CPZ-containing diet(CPZ, *n*=5-6/genotype) for four weeks. (A,B) Iba1 protein level inthe CC region was assessed by Western blot (WB) analysis, and the fold change (CPZ vs. CTL) wasquantified. (C) A negative correlation was observed betweenthe fold change of Iba1^+^ microglia (A) and the fold change of dMBP^+^myelin debris upon CPZ treatment. Valuesare mean ± SEM. One-way ANOVA. ** *P*< 0.01.

**Additional file 2: Figure sup 2.** Lack of effects of cuprizone-induceddemyelination on the blood-brainbarrier integrity among apoE-TR mice. (A) Brain samples ofapoE-TR mice from CPZ-treated group (*n*=16/genotype) were subjected toimmunofluorescence staining for CLDN5(a tight junction-associated protein) and Glut1 (vascular marker). Representative images of CLDN5 (Green) staining and Glut1 (Red)staining in the CC region of apoE-TR mice are shown. Scale bar, 50 µm. Values are mean ± SEM. One-way ANOVA.N.S. not significant.

**Additional file 3: Figure sup 3.** Similar levels of astrogliosis were observed in apoE-TR mice upon cuprizone-induced demyelination. The astrogliosis in the CC area of CTL (*n*=5/genotype) and CPZ-treated (*n*=5-6/genotype) apoE-TR mice was examined by immunostaining for GFAP. (A) Representative images of GFAP^+^astrocytes in the CC area of apoE-TR mice are shown. Scale bar, 100 µm. (B) The morphology of GFAP^+^astrocytes in apoE-TR mice are shown. Scale bar, 25 µm. (C) The fold change of GFAP^+^ staining in CTL (*n*=5/genotype) and CPZ-treated (*n*=5-6/genotype) apoE-TR mice was quantified. (D) The number of GFAP^+^ astrocyte per high-power field (HPF; 3 HPFs/mouse) was analyzed. (E) The surface area of GFAP^+^ astrocyte was quantified. Values are mean ± SEM. Two-way ANOVA. N.S., not significant.

**Additional file 4: Figure sup 4.** Transcriptional changes of selected astrocyte-enrichedgenes in apoE-TR mice upon cuprizone-induced demyelination. The CC region of apoE-TR mice treated with normal or CPZ diet was subjected to transcriptomic profiling. Heat map showing transcriptional changes (CPZ vs CTL groups) of selected astrocyte-enriched genes identified from RNA-Seq. The scaled expression value (row Z score) is shown with a blue-red color scheme, denoting red as higher expression, and blue as lower expression.

**Additional file 5: Figure sup 5.** Transcriptional changes of selected immuneresponse-related genes in apoE-TR mice upon cuprizone-induced demyelination.RNA was extracted from the CC area of CTL (n=5/genotype) and CPZ-treated (*n*=5-6/genotype) apoE-TR mice. (A-F)The expression of immune response-associated genes (i.e., *Cst7*, *Ccl2*, *Csf1*, *Csf1r*, *Serpina3n*, and *Tnf-α*) wasmeasured by real-time PCR. Values are mean ± SEM. Mann-Whitney tests followedby Bonferroni correction for multiple comparisons were used. *P < 0.0167;N.S. not significant.

**Additional file 6: Figure sup 6.** The key molecules that regulate microglialfunctions are up-regulated in an apoE isoform-dependent manner uponcuprizone-induced demyelination. RNA was extracted from the CC area of CTL(*n*=5/genotype) and CPZ-treated (*n*=5-6/genotype) apoE-TR mice. (A-H) The expression of genes relatedto microglial activation, inflammation, and lipid sensing (*Trem2, Tyrobp,Aim2, Btk, B2m, Ctsd, C3,* and *Cr3*) was analyzed by real-time PCR. (I-L) The expression of genes involvedin microglial migration (*Mmp2* and *Mmp12*) and phagocytosis (*Axl*)were examined by real-time PCR. Values are mean ± SEM. Two-way ANOVA. * *P* <0.05; ** *P* < 0.01. N.S., not significant.

## Data Availability

All data generated in this study are included in this published article. Raw RNA-seq data (fastq files) has been deposited in the Synapse with the dataset identifier https://www.synapse.org/#!Synapse:syn27717736.

## Abbreviations

AD: Alzheimer’s disease
CNS: Central nervous system
LBD: Lewy body dementia
CC: Corpus callosum
TR: Targeted replacement
apoE: Apolipoprotein E
DAM: Disease associated microglia
TREM2: Triggering Receptor Expressed on Myeloid cells-2
q-PCR: Quantitative real-time PCR
WB: Western blotting
IHC: Immunohistochemistry
IF: Immunofluorescence
CPM: Count per million
TMM: Trimmed mean of M-values normalization
FDR: False discovery rate
FC: Fold change
PFN: Planar Filtered Network
BBB: Blood–brain barrier
MBP: Myelin basic protein
dMBP: Degraded myelin basic protein
HPF: High-power field
GFAP: Glial fibrillary acidic protein
DEGs: Differentially expressed genes
Csf1: Colony-stimulating factor 1
Mcp-1: Monocyte chemoattractant protein 1
MEGENA: Multiscale embedded gene co-expression network analysis
Plin2: Perilipin 2
TEM: Transmission electron microcopy
