# Supplementary figures and images for: Opposing effects of apoE2 and apoE4 on microglial activation and lipid metabolism in response to demyelination
Source: Mol Neurodegener. 2022 Nov 23;17:75. doi: 10.1186/s13024-022-00577-1 (PMC9682675; doi:10.1186/s13024-022-00577-1)

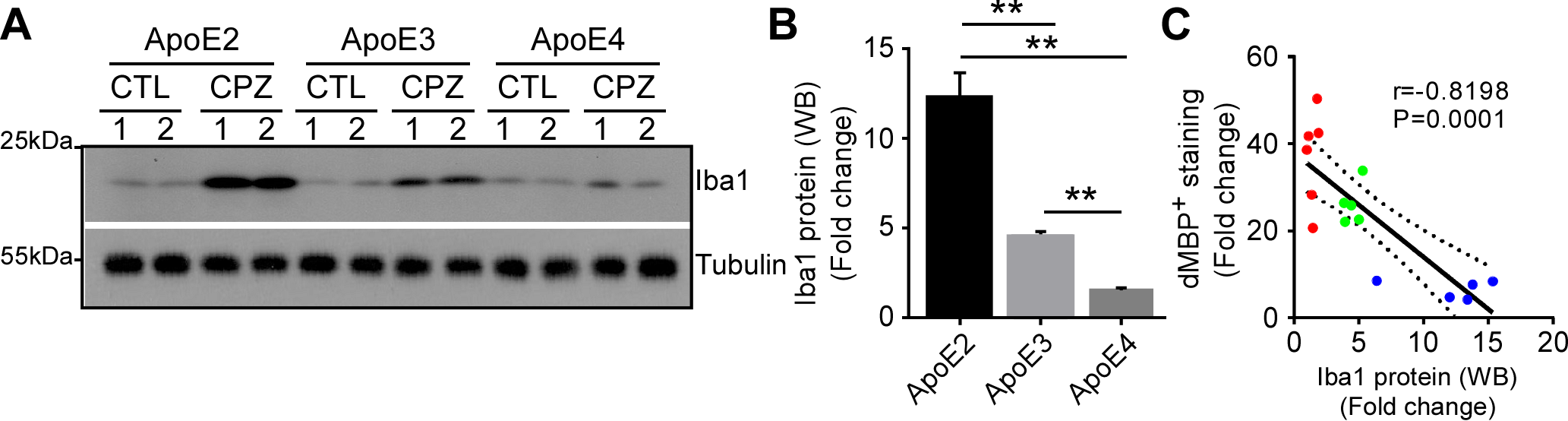

Supplement: Supplementary file 1 — Additional file 1: Figure sup 1. Opposing effects of apoE2 and apoE4 on Iba1 proteinlevels and their correlation with the amount of myelin debris in the corpuscallosum of apoE-TR mice upon cuprizone-induced demyelination. ApoE-TR micewere fed with either normal diet (CTL, n=5/genotype), or CPZ-containing diet(CPZ, n=5-6/genotype) for four weeks. (A,B) Iba1 protein level inthe CC region was assessed by Western blot (WB) analysis, and the fold change (CPZ vs. CTL) wasquantified. (C) A negative correlation was observed betweenthe fold change of Iba1+ microglia (A) and the fold change of dMBP+myelin debris upon CPZ treatment. Valuesare mean ± SEM. One-way ANOVA. ** P< 0.01. [file 13024_2022_577_MOESM1_ESM.tif]

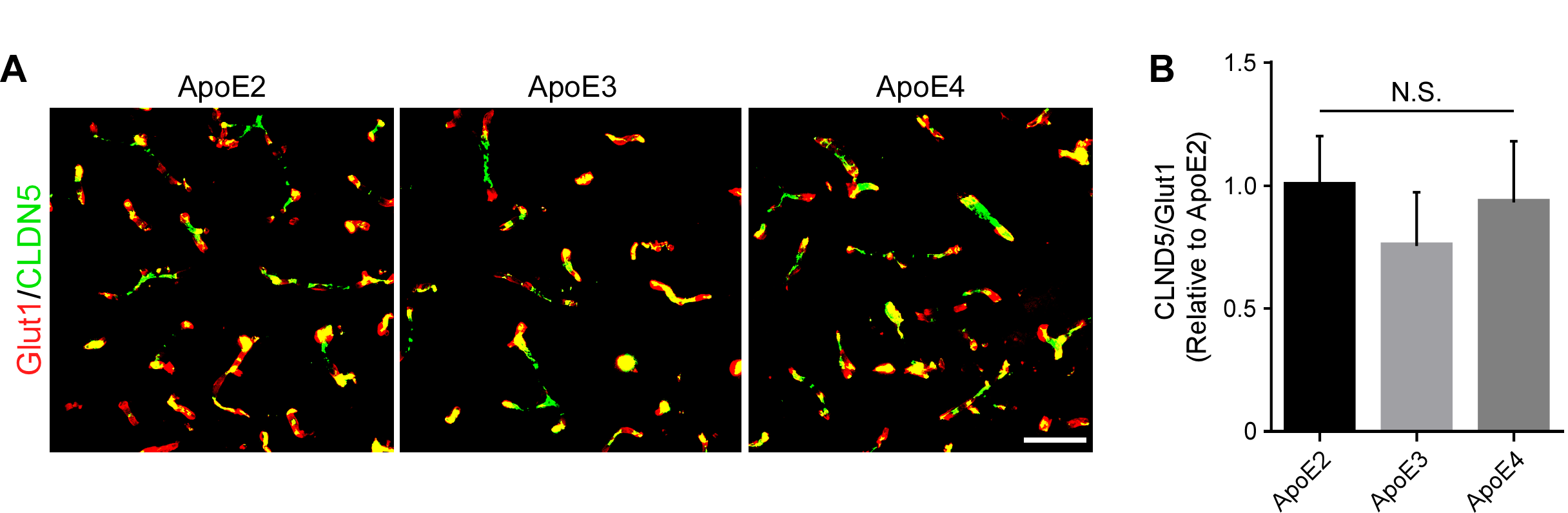

Supplement: Supplementary file 2 — Additional file 2: Figure sup 2. Lack of effects of cuprizone-induceddemyelination on the blood-brainbarrier integrity among apoE-TR mice. (A) Brain samples ofapoE-TR mice from CPZ-treated group (n=16/genotype) were subjected toimmunofluorescence staining for CLDN5(a tight junction-associated protein) and Glut1 (vascular marker). Representative images of CLDN5 (Green) staining and Glut1 (Red)staining in the CC region of apoE-TR mice are shown. Scale bar, 50 µm. Values are mean ± SEM. One-way ANOVA.N.S. not significant. [file 13024_2022_577_MOESM2_ESM.tif]

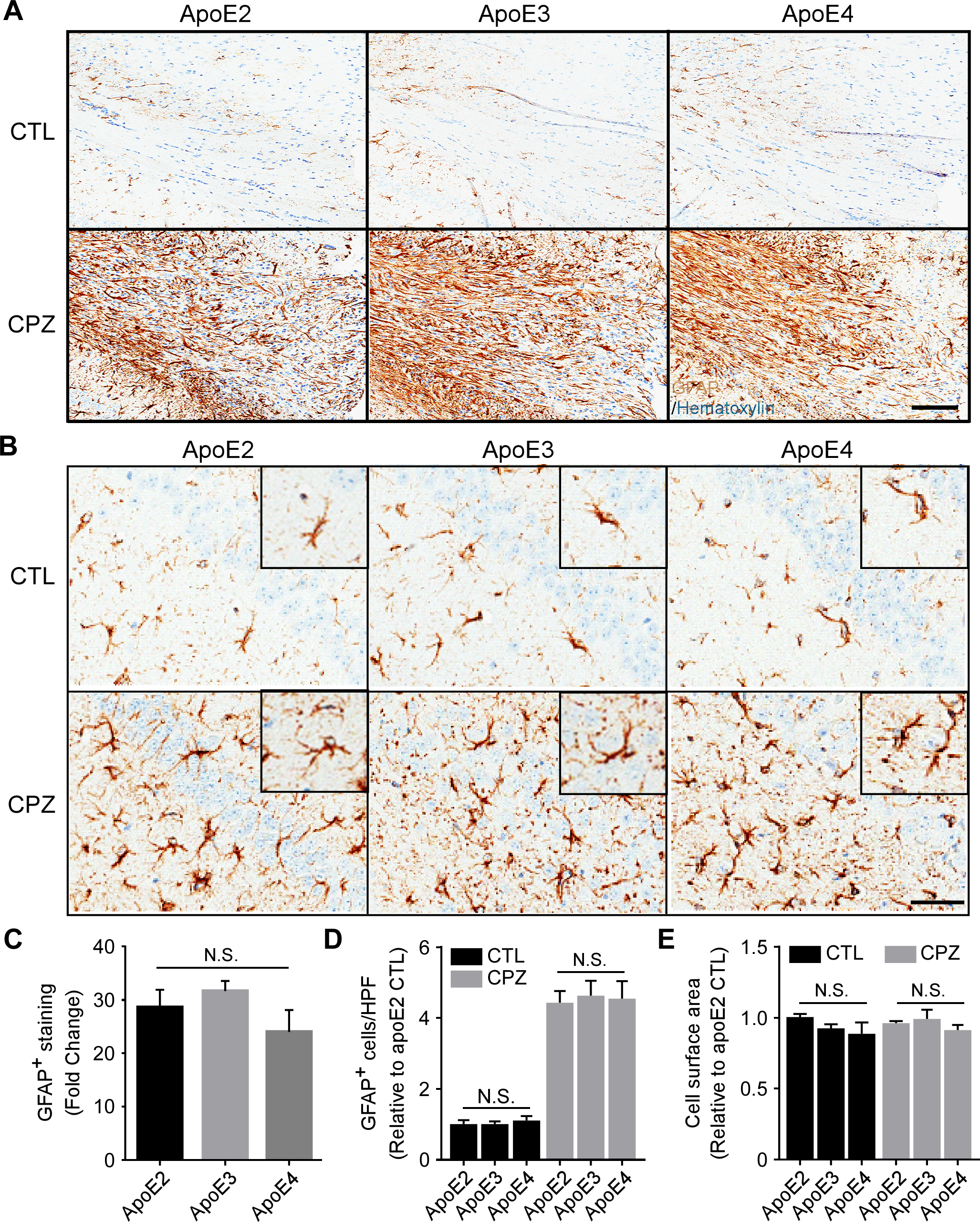

Supplement: Supplementary file 3 — Additional file 3: Figure sup 3. Similar levels of astrogliosis were observed in apoE-TR mice upon cuprizone-induced demyelination. The astrogliosis in the CC area of CTL (n=5/genotype) and CPZ-treated (n=5-6/genotype) apoE-TR mice was examined by immunostaining for GFAP. (A) Representative images of GFAP+astrocytes in the CC area of apoE-TR mice are shown. Scale bar, 100 µm. (B) The morphology of GFAP+astrocytes in apoE-TR mice are shown. Scale bar, 25 µm. (C) The fold change of GFAP+ staining in CTL (n=5/genotype) and CPZ-treated (n=5-6/genotype) apoE-TR mice was quantified. (D) The number of GFAP+ astrocyte per high-power field (HPF; 3 HPFs/mouse) was analyzed. (E) The surface area of GFAP+ astrocyte was quantified. Values are mean ± SEM. Two-way ANOVA. N.S., not significant. [file 13024_2022_577_MOESM3_ESM.tif]

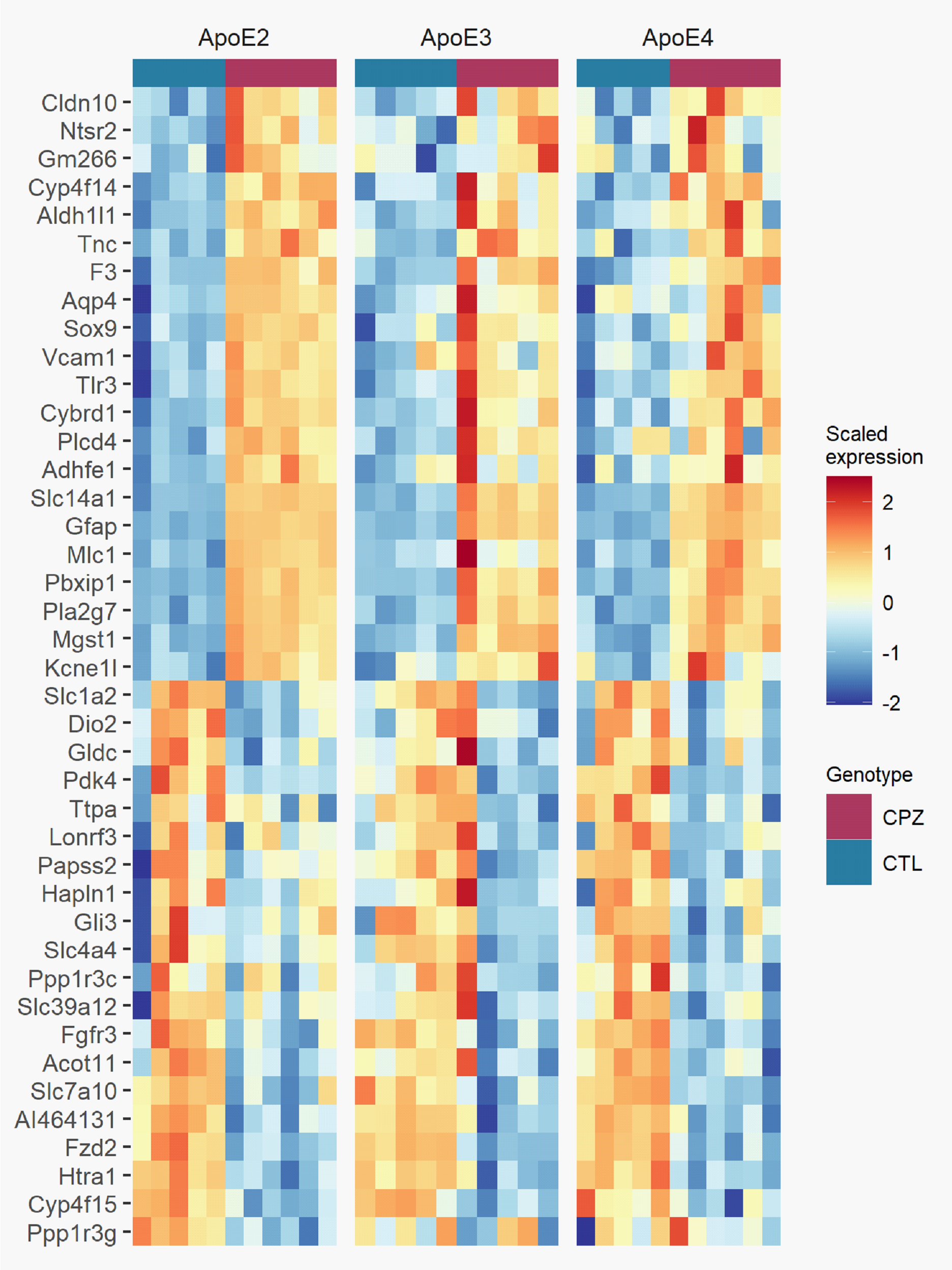

Supplement: Supplementary file 4 — Additional file 4: Figure sup 4. Transcriptional changes of selected astrocyte-enrichedgenes in apoE-TR mice upon cuprizone-induced demyelination. The CC region of apoE-TR mice treated with normal or CPZ diet was subjected to transcriptomic profiling. Heat map showing transcriptional changes (CPZ vs CTL groups) of selected astrocyte-enriched genes identified from RNA-Seq. The scaled expression value (row Z score) is shown with a blue-red color scheme, denoting red as higher expression, and blue as lower expression. [file 13024_2022_577_MOESM4_ESM.tif]

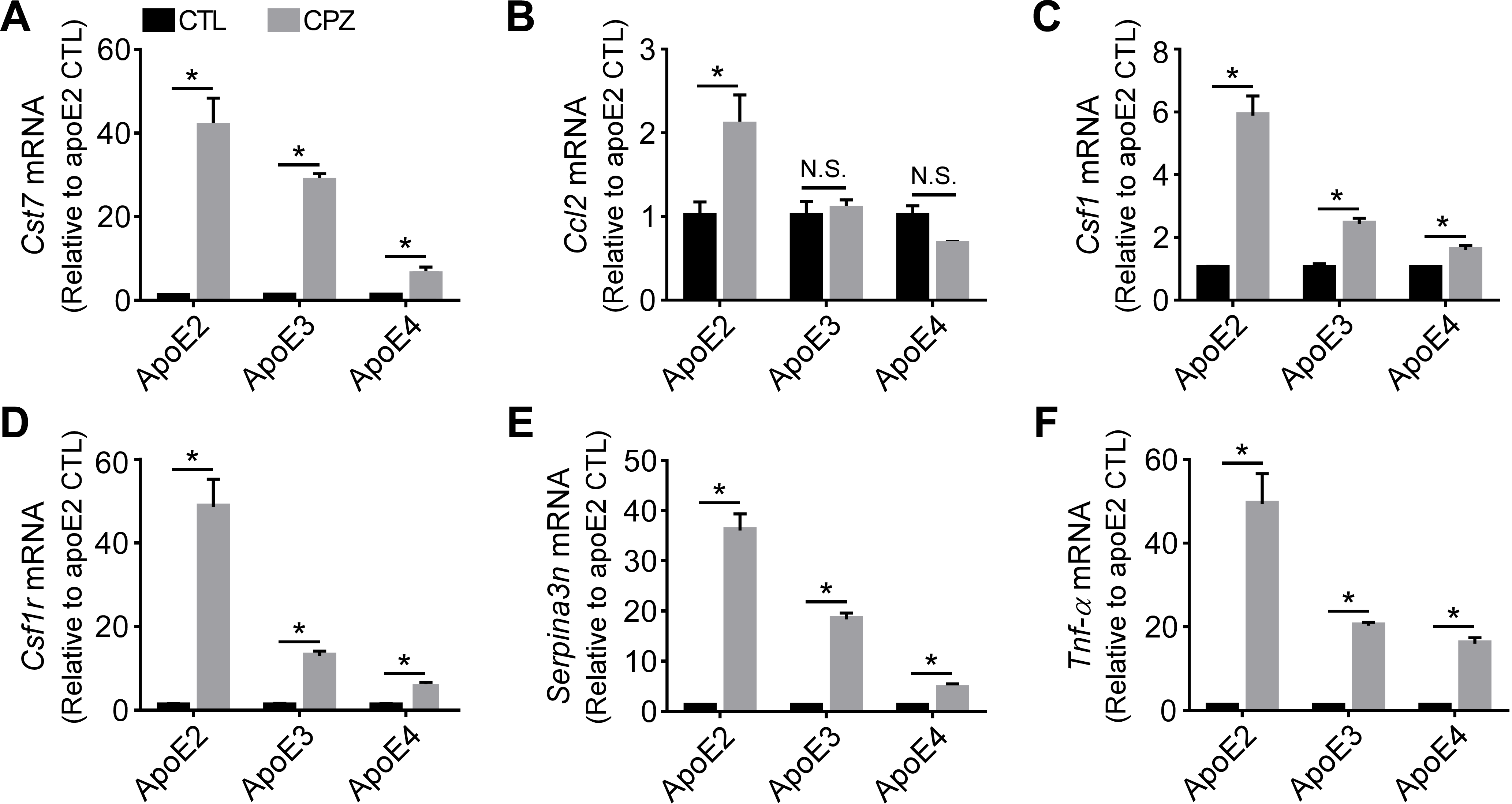

Supplement: Supplementary file 5 — Additional file 5: Figure sup 5. Transcriptional changes of selected immuneresponse-related genes in apoE-TR mice upon cuprizone-induced demyelination.RNA was extracted from the CC area of CTL (n=5/genotype) and CPZ-treated (n=5-6/genotype) apoE-TR mice. (A-F)The expression of immune response-associated genes (i.e., Cst7, Ccl2, Csf1, Csf1r, Serpina3n, and Tnf-α) wasmeasured by real-time PCR. Values are mean ± SEM. Mann-Whitney tests followedby Bonferroni correction for multiple comparisons were used. *P < 0.0167;N.S. not significant. [file 13024_2022_577_MOESM5_ESM.tif]

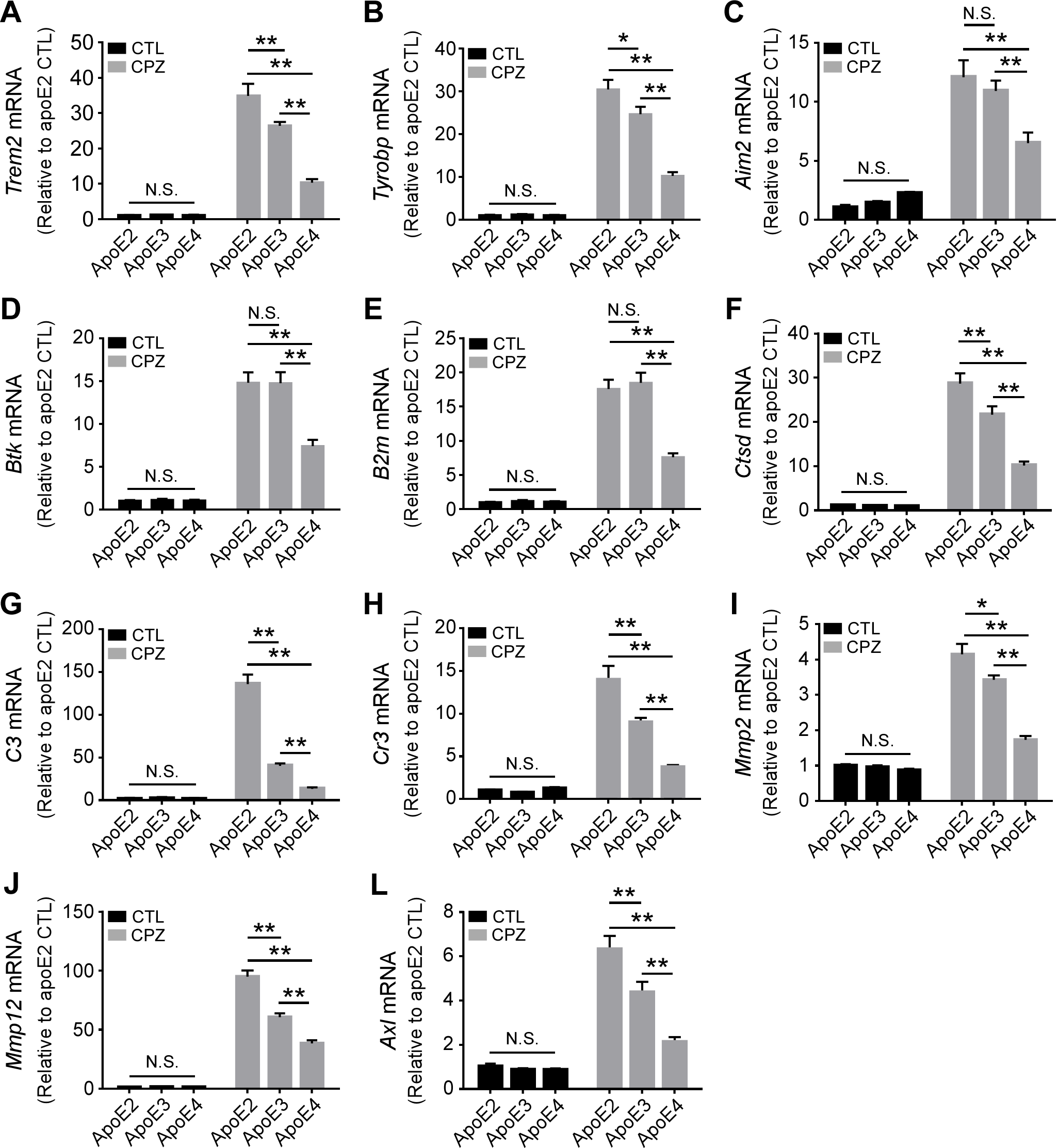

Supplement: Supplementary file 6 — Additional file 6: Figure sup 6. The key molecules that regulate microglialfunctions are up-regulated in an apoE isoform-dependent manner uponcuprizone-induced demyelination. RNA was extracted from the CC area of CTL(n=5/genotype) and CPZ-treated (n=5-6/genotype) apoE-TR mice. (A-H) The expression of genes relatedto microglial activation, inflammation, and lipid sensing (Trem2, Tyrobp,Aim2, Btk, B2m, Ctsd, C3, and Cr3) was analyzed by real-time PCR. (I-L) The expression of genes involvedin microglial migration (Mmp2 and Mmp12) and phagocytosis (Axl)were examined by real-time PCR. Values are mean ± SEM. Two-way ANOVA. * P <0.05; ** P < 0.01. N.S., not significant. [file 13024_2022_577_MOESM6_ESM.tif]
